# Self-Healing Injectable Hydrogels for Tissue Regeneration

**DOI:** 10.1021/acs.chemrev.2c00179

**Published:** 2022-08-05

**Authors:** Pascal Bertsch, Mani Diba, David J. Mooney, Sander C. G. Leeuwenburgh

**Affiliations:** † Department of Dentistry-Regenerative Biomaterials, Radboud Institute for Molecular Life Sciences, Radboud University Medical Center, 6525 EX Nijmegen, The Netherlands; ‡ John A. Paulson School of Engineering and Applied Sciences, Harvard University, Cambridge, Massachusetts 02138, United States; § Wyss Institute for Biologically Inspired Engineering at Harvard University, Boston, Massachusetts 02115, United States

## Abstract

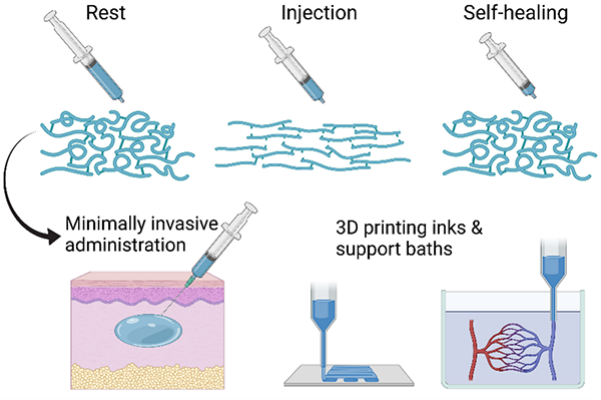

Biomaterials with the ability to self-heal and recover their structural integrity offer many advantages for applications in biomedicine. The past decade has witnessed the rapid emergence of a new class of self-healing biomaterials commonly termed injectable, or printable in the context of 3D printing. These self-healing injectable biomaterials, mostly hydrogels and other soft condensed matter based on reversible chemistry, are able to temporarily fluidize under shear stress and subsequently recover their original mechanical properties. Self-healing injectable hydrogels offer distinct advantages compared to traditional biomaterials. Most notably, they can be administered in a locally targeted and minimally invasive manner through a narrow syringe without the need for invasive surgery. Their moldability allows for a patient-specific intervention and shows great prospects for personalized medicine. Injected hydrogels can facilitate tissue regeneration in multiple ways owing to their viscoelastic and diffusive nature, ranging from simple mechanical support, spatiotemporally controlled delivery of cells or therapeutics, to local recruitment and modulation of host cells to promote tissue regeneration. Consequently, self-healing injectable hydrogels have been at the forefront of many cutting-edge tissue regeneration strategies. This study provides a critical review of the current state of self-healing injectable hydrogels for tissue regeneration. As key challenges toward further maturation of this exciting research field, we identify (i) the trade-off between the self-healing and injectability of hydrogels vs their physical stability, (ii) the lack of consensus on rheological characterization and quantitative benchmarks for self-healing injectable hydrogels, particularly regarding the capillary flow in syringes, and (iii) practical limitations regarding translation toward therapeutically effective formulations for regeneration of specific tissues. Hence, here we (i) review chemical and physical design strategies for self-healing injectable hydrogels, (ii) provide a practical guide for their rheological analysis, and (iii) showcase their applicability for regeneration of various tissues and 3D printing of complex tissues and organoids.

## 1 Introduction

Biomaterials are designed to support, treat, augment, repair, or replace a part of body tissue or its function. Over the past decades, the steady rise and optimization of biomaterials has revolutionized many fields of medicine.^1,2^ Biomaterials are subjected to continuous mechanical load or biochemical degradation which can impair their structural integrity and ultimately their functionality. Hence, extensive research efforts have been dedicated to the design of biomaterials that are self-healing, i.e., can halt or even reverse damages induced by mechanical or biochemical stresses. While self-healing may refer to the recovery of any biomaterial function, it most commonly describes the recovery of a material’s structural integrity and associated mechanical properties.^3−8^ This review provides an overview of self-healing injectable hydrogels, a particular class of self-healing biomaterials that can fluidize under shear stress followed by recovery of their mechanical properties, and show exciting prospects for applications in tissue regeneration and 3D (bio)printing.

In context of self-healing, hydrogels are a promising class of biomaterials because of their dynamic and diffusive nature compared to traditional polymers, ceramics, or cements. Hydrogels with self-healing capacity can be assembled from a large toolbox of biocompatible materials exploiting noncovalent or dynamic covalent interactions.^9−13^ Hydrogels have long been recognized as promising biomaterial platforms with various applications in biomedicine.^14−16^ Hydrogels can be engineered to closely resemble the natural 3D environment, distribution of cell ligands and nutrients, and viscoelasticity of the extracellular matrix (ECM) of various tissues.^17−19^ They are common scaffold materials for the ex situ cultivation of tissue or organoids, commonly known as tissue engineering.^20−22^ Hydrogels are also increasingly used in cell culture, particularly in 3D, which has resolved many issues such as abnormal cell shaping or differentiation observed for cells cultured in 2D monolayers or on hard substrates.^23−25^ Hydrogel-based cell culture is still in its infancy, and several groups have provided practical guides on the use of hydrogels for cell culture to promote its implementation in biomedical research.^26−29^

**Figure 1:**
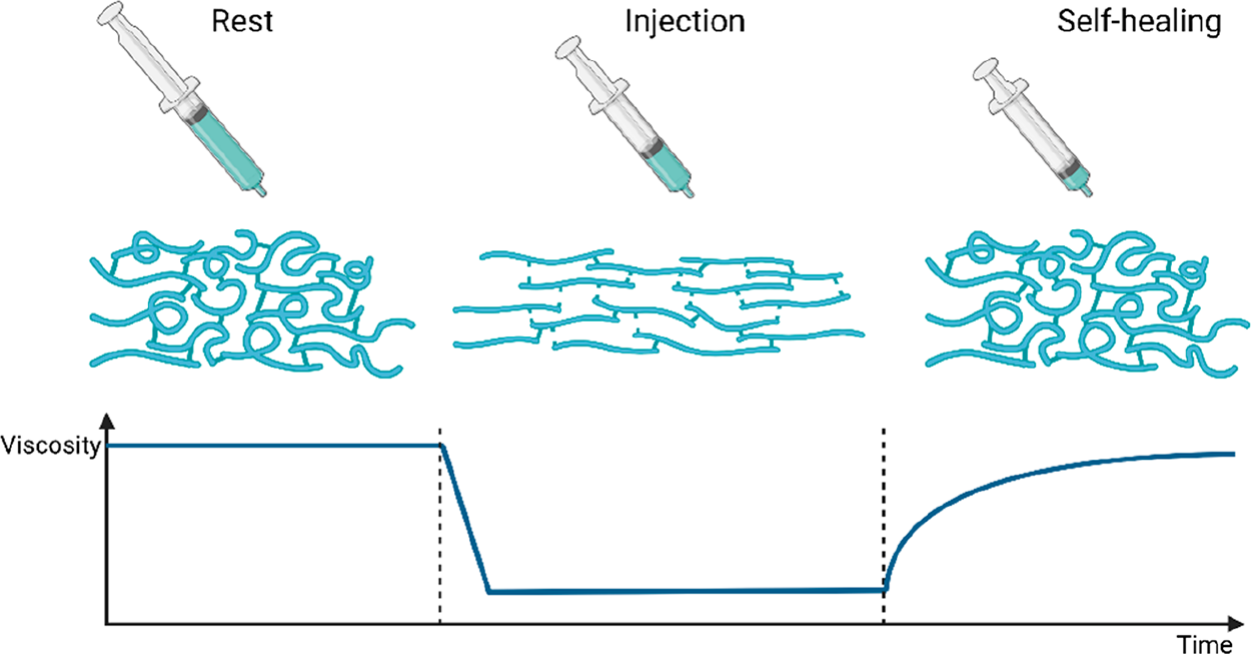
Schematic behavior of a self-healing injectable hydrogel with (i) gel-like properties at rest, (ii) fluidization under shear due to reversible chemistry and/or alignment in the flow field, and (iii) self-healing of the original structure and mechanical properties after flow.

One particularly interesting class of self-healing hydrogels involves injectable or printable (in context of 3D printing) hydrogels. These self-healing injectable hydrogels are able to temporarily fluidize under shear stress and recover their original structure and mechanical properties after release of the applied stress, as schematically illustrated in Figure 1. This unique feature of self-healing injectable hydrogels has paved the way for several exciting applications in biomedicine, as summarized in Box 1. Most importantly, self-healing injectable hydrogels can be administered in a minimally invasive manner at the target site.^30−32^ The hydrogels can be structurally and mechanically fine-tuned to mimic various tissues and aid regeneration by providing mechanical tissue support.^33,34^ Because of their rapid self-healing, these hydrogels can be administered with high spatial control and mold into patient-specific tissue defects without undesired off-target leakage. This opens many avenues for personalized interventions, in particular in combination with noninvasive imaging technqiues.^35−38^ Self-healing injectable hydrogels can be exploited for locally targeted and sustained delivery of therapeutics,^39−42^ and their shear-thinning plug flow in syringes facilitates administration of live cells.^43−45^ Ultimately, self-healing injectable hydrogels are increasingly used as inks in 3D (bio)printing or support matrices in freeform 3D printing, and facilitate printing of complex tissue models and organoids with spatial control over material composition and distribution of cells or biomolecules.^46−49^

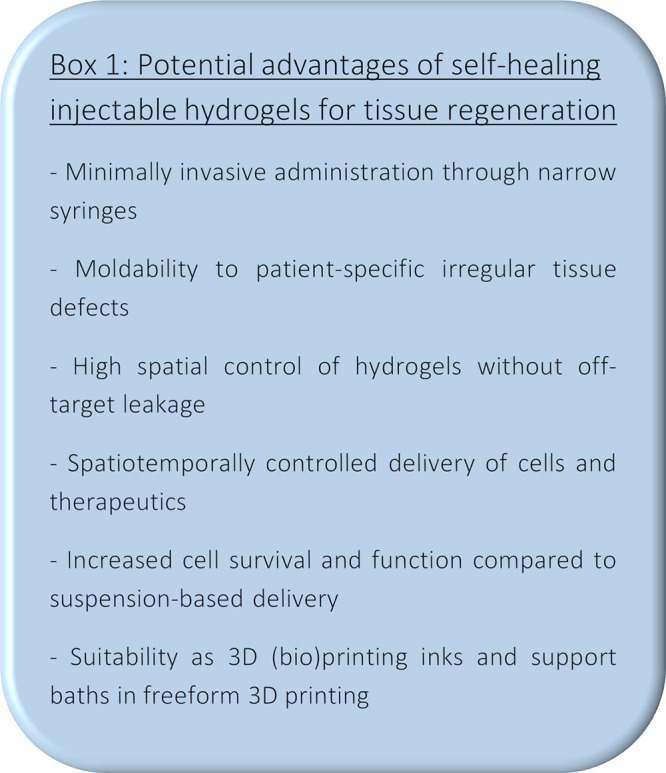



Accordingly, there has been an increasing number of studies reporting novel tissue regeneration strategies exploiting self-healing injectable hydrogels. Nevertheless, there are still several bottlenecks that currently impede their development and translation. First of all, the nomenclature and definitions in literature are often inconsistent, wherefore we summarized the relevant terms related to self-healing injectable hydrogels in section 2. The design of self-healing injectable hydrogels requires precise control over hydrogel properties that allow for injectability, self-healing, as well as desired tissue interactions. Hence, we provide an overview of chemical and physical design strategies in section 3. There is currently no clear consensus which rheological protocols and benchmarks are most suitable for the characterization of self-healing injectable hydrogels. In section 4, we provide a practical guide for their rheological analysis. The translation of self-healing injectable hydrogels toward biomedical applications requires hydrogels designed specifically for the target tissue and mode of regeneration. Consequently, section 5 elaborates hydrogel requisites and regeneration strategies for specific tissues. Ultimately, self-healing injectable hydrogels are widely used as 3D (bio)printing inks, but the lack of quantitative parameters associated with printability often impedes their translation from the rheometer to the printer. In section 6, we discuss the requirements for hydrogels as 3D (bio)printing inks and their potential for printing complex tissues and organoids.

## 2 The Self-Healing Dictionary

The field of self-healing biomaterials is still in its infancy with first reports dating from the late 1990s^50,51^ but has evolved very rapidly, particularly during the past decade. Upon emergence of self-healing biomaterials as a research field, various terms and nomenclatures have been introduced which are not always well-defined and sometimes used inconsistently in literature. In 2011, Brochu et al.^3^ introduced a classification of biomaterials depending on their self-healing capacity. *Zeroth generation* materials are thereafter able to merely retard deterioration, mostly by tailored composite mixtures that reduce wear or crack propagation.^52^*First generation* materials are able to irreversibly repair defects without restoring the original structure, mostly by healing agents incorporated in capsules or tubes that are released upon breakage, referred to as **extrinsic** self-healing.^53,54^*Second generation* materials are able to fully restore their original structure due to noncovalent or reversible covalent chemistry, also called **intrinsic** self-healing.^4,9^ This may occur spontaneously under ambient conditions (**autonomous**) or rely on an external trigger such as heat or UV-light (**stimulus**).^15,55^ This traditional classification of self-healing focuses on the avoidance and repair of small defects induced by physicochemical deterioration of biomaterials in situ. However, there has been an increasing demand for biomaterials that are able to fully fluidize temporarily, i.e., exhibit a shear-induced viscosity decrease by the order of decades, followed by self-healing and recovery of their original mechanical properties.^12,30^ This self-healing injectable behavior is almost exclusively observed for viscoelastic soft matter such as hydrogels with reversible chemistry, along with certain dense suspensions, colloidal glasses and gels, and emulsions.^56−62^ The driving force for this development has been the ambition to administer biomaterials in a minimally invasive manner, along with the rapid emergence of 3D printing. Because of their ability to fully fluidize during capillary extrusion in a syringe or 3D printing nozzle, these hydrogels are commonly termed **injectable**, or in context of 3D printing, **printable**. Hence, self-healing injectable hydrogels unite two seemingly contradictory properties, flowability and mechanical stability, depending on the experienced shear stress. Compared to other self-healing biomaterials, they experience much higher stresses, span much wider ranges of viscosity and need to recover at much shorter time scales. In continuation of the classification introduced by Brochu et al.,^3^ we propose that self-healing injectable biomaterials represent the *third generation* of self-healing biomaterials. The relevant terms used for self-healing biomaterials are summarized in Box 2. An extensive compilation of terminology used for biomaterials is provided by Vert et al.^63^

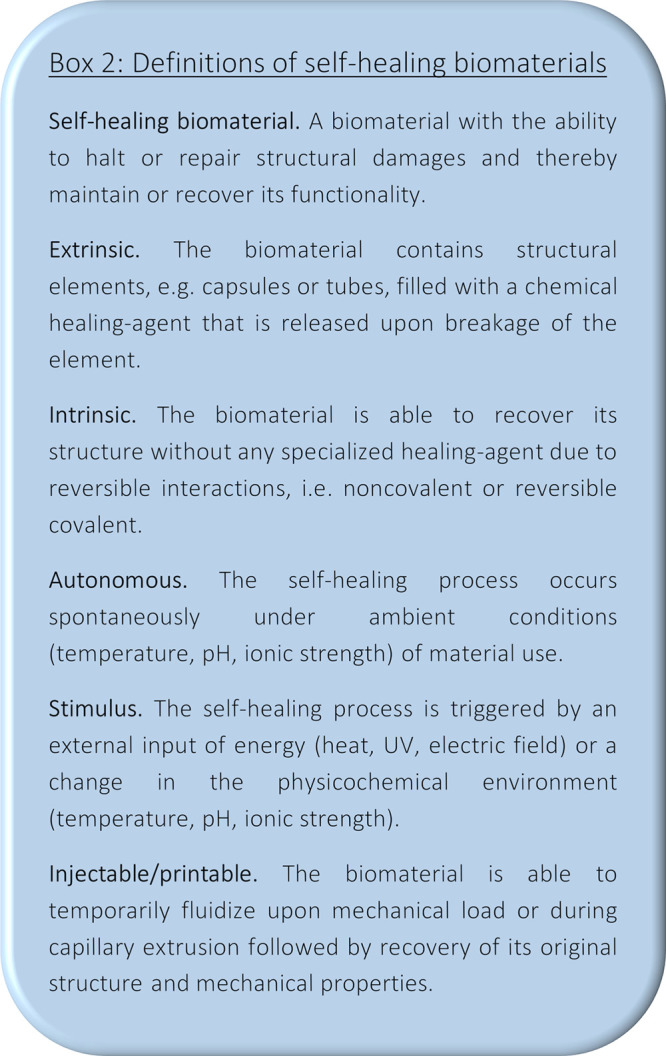



## 3 Design Strategies for Self-Healing Injectable Hydrogels

Hydrogel formation requires the establishment of cross-links between molecular and/or particulate building blocks in an aqueous environment, resulting in assembly of these units into a 3D interconnected hydrogel network. A wide range of material types have been used as building blocks for the formation of self-healing injectable hydrogels. Early work on reversible hydrogels has focused particularly on the reaction of boric acid with charged polysaccharides.^64−67^ Natural polymers commonly used for the formation of such hydrogels include gelatin, collagen, hyaluronic acid, alginate, and chitosan, while synthetic polymers include poly(ethylene glycol) (PEG) and poly(*N*-isopropylacrylamide). These different material systems can be functionalized with various reversible cross-linking strategies. Evidently, hydrogels and cross-linking strategies envisioned for biomedical applications should be biocompatible, i.e., (i) should not trigger an excessive inflammatory response, (ii) be biodegradable within a desired time frame (unless envisioned as permanent implant), and (iii) be cleared from the body without the production of toxic byproducts. Also, it is important to bear in mind that hydrogels may be affected by swelling, hysteresis, or aging in situ, which can affect their mechanical and self-healing properties.^68−70^

The design of hydrogels that can temporarily fluidize during extrusion and exhibit rapid self-healing requires in-depth understanding of chemical mechanisms involved in hydrogel formation. A fundamental prerequisite for chemical design of such material systems is the reversibility of bonds responsible for cross-linking of the hydrogel network. The reversibility of interactions is a key design criteria that allows for injectability as well as *intrinsic* self-healing (see section 2). Additionally, the physical form and network structure of hydrogels can strongly impact their properties, including injectability and self-healing performance. Therefore, in this section, we discuss key strategies for the design of self-healing injectable hydrogels both in terms of chemical mechanism (section 3.1) as well as physical form (section 3.2).

### 3.1 Chemical Design of Self-Healing Injectable Hydrogels

The design of hydrogels that are self-healing and injectable but also physically stable is a complex trade-off that needs to be carefully balanced. On one hand, the strength and density of cross-links is crucial for the formation of mechanically stable hydrogels. On the other hand, the self-healing and injection capacity of hydrogels largely depend on the reversible nature of the cross-links. Consequently, the chemical design of self-healing injectable hydrogels is achieved by exploiting noncovalent interactions, dynamic covalent bonds, or combinations thereof. The dynamic behavior of these reversible cross-links is regulated by their association rate constant (*k*_a_), dissociation rate constant (*k*_d_), and equilibrium binding constant (*K*_eq_ = *k*_a_/*k*_d_).^71^*k*_a_ determines the rate of cross-link formation, and higher *k*_a_ values translate to faster gelation kinetics and self-healing, e.g., after shear-induced damage during injection.^72,73^*k*_d_ on the other hand indicates the rate of rupture of these cross-links. Higher *k*_d_ values can translate to higher molecular mobility in the gel network and therefore favor injectability and a larger degree of self-healing after mechanical damage. *K*_eq_, which is determined by the ratio between *k*_a_ and *k*_d_, is consequently related to all these factors (i.e., gelation kinetics, injectability, and self-healing).^71,72,74^ An overview of *K*_eq_ of interactions commonly employed for self-healing injectable hydrogels is provided in Table 1. Generally, excessively large *K*_eq_ values result in hydrogels that are not self-healing nor injectable due to a low dynamicity of the cross-links. Lower *k*_eq_ values favor injectability and self-healing but may result in hydrogels with lack of integrity and rapid dissolution.^72,73^

**Table 1:** Overview of Equilibrium Binding Constants *K*_eq_ of Reversible Interactions Commonly Employed for Self-Healing Injectable Hydrogels

noncovalent interactions					
type	metal coordination	metal coordination	metal coordination	hydrophobic	hydrogen bonding
motif	catechol-Fe^3+^	bisphosphonate-Ca^2+^	histidine-Zn^2+^	β-cyclodextrin-adamantane host–guest complexation	ureidopyrimidinone
*K*_eq_ (M^–1^)	10^40^–10^45^	10^17.25^	10^6.5^	10^5^	6 × 10^7^
conditions	in aqueous media	in aqueous media at 25 °C	in aqueous media at 25 °C	in aqueous media	in chloroform at 25 °C
refs	(72,79)	(72,80)	(72,81)	(82,83)	(84)

dynamic covalent bonds		
reaction	Diels–Alder	Schiff base
*K*_eq_ (M^–1^)	∼10°	10^4^–10^6^
conditions	for furan and maleimide motifs; In aqueous media at ∼92.5 °C	for hydrazone linkages
refs	(71,85)	(86,87)

Hence, it is important to tune the dynamics of self-healing hydrogels to the relevant processing time scales. Thereto, the cross-link lifetime (τ_b_ = 1/*k*_d_), which is the average time that a bond is at its associated state, can be a useful measure.^71,75,76^ Previous reports indicate that hydrogels with τ_b_ values in a range of 1 μs to 1 min exhibit self-healing at relevant time scales.^77,78^ However, a purely chemical approach at self-healing via binding constants may not be sufficient to characterize self-healing hydrogels, which also involves many aspects of polymer physics. In practice, rheology provides a useful tool to determine self-healing kinetics of hydrogels. For ideal Maxwell fluids, relating the material relaxation time τ_R_ to the relevant observation time τ_obs_ of a specific process, i.e., the Deborah number De = τ_R_/τ_obs_, provides a good measure.^71^ As many hydrogels behave as elastic solids before injection and no relaxation time can be determined, measuring their transient self-healing kinetics is a viable alternative. The rheological characterization of hydrogels will be discussed in detail in section 4.

Consequently, there is no “one-size-fits-all” chemical design strategy for self-healing injectable hydrogel systems, and the selected strategy requires various interrelated considerations that depend on the specific hydrogel application. In the following sections, we discuss key chemical strategies for the design of self-healing injectable hydrogels for tissue regeneration.

#### 3.1.1 Noncovalent Interactions

**Figure 2:**
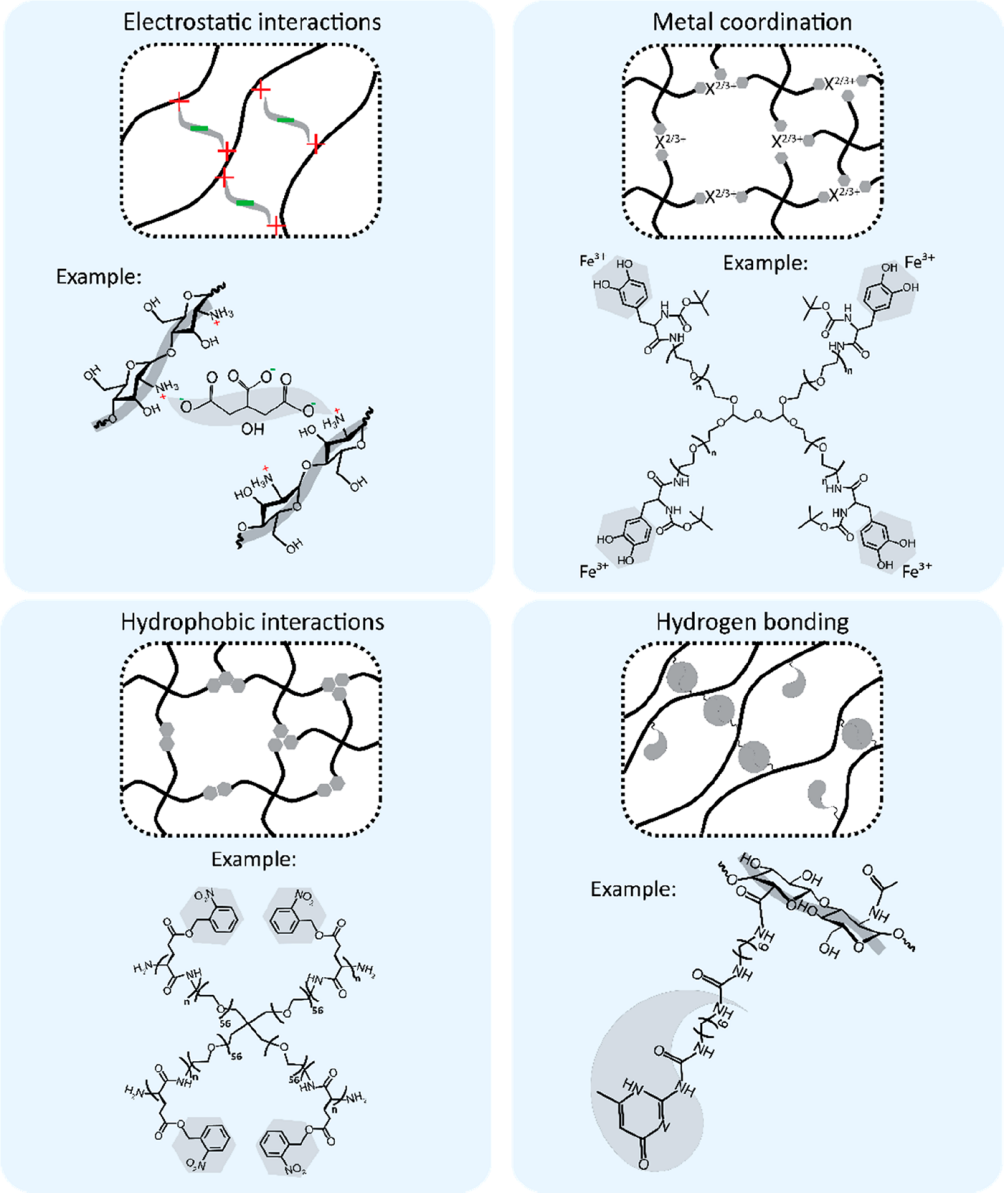
Overview of common noncovalent chemical interactions used for the design of self-healing injectable hydrogels. Examples for electrostatic, metal coordination, hydrophobic, and hydrogen bonding are based on refs (91), (92), (32), and (93), respectively. Adapted with permission from ref (4). Copyright 2018 Wiley.

Noncovalent interactions are widespread in nature, as their versatility allows for unique material behavior, ranging from soft and short-lived hagfish slime to the strong adhesive capacity of marine mussels.^88−90^ Thanks to their reversibility and possibility to be employed in aqueous environments, various noncovalent interaction strategies have been used for the design of self-healing injectable hydrogels, as summarized in Figure 2.

##### 3.1.1.1 Electrostatic Interactions

Electrostatic attractive forces between oppositely charged chemical groups have been exploited in a wide range of strategies for the design of self-healing hydrogels. One common route for this design is the use of intrinsic attractive forces between anionic and cationic polymers. Examples for such strategies include combinations of anionic (bovine) and cationic (porcine) gelatin.^62^ Alternatively, an ionic monomer such as negatively charged citrate can be added as a cross-linker to a cationic polymer such as chitosan to form self-healing injectable hydrogels.^91^ Various natural polymers like proteins are polyampholytes, and both anionic and cationic groups exist along their chains. Consequently, “anionic” or “cationic” designations only refer to the net charge of such macromolecules. Synthetic zwitterionic polymers can be developed by copolymerization of anionic and cationic building blocks, which can assemble into self-healing hydrogels based on intermolecular electrostatic attractive forces.^94,95^ Importantly, electrostatic interactions are highly susceptible to the ionic strength and pH of their environment. Under conditions of high ionic strength electrostatic forces are screened and might not be sufficiently strong to form hydrogels or maintain their physical integrity.^96^ Similarly, changes in pH can alter the net charge of polymers, potentially reverting electrostatic attractive forces to repulsive interactions.^91^ In spite of the potential negative impact of these environmental factors, they can also provide unique opportunities for stimulus-responsive control of in situ assembly and tuning of hydrogel properties.^61,97−99^

##### 3.1.1.2 Metal Coordination Interactions

Metal–ligand coordination interactions are widely employed in nature for structural support. These interactions typically take place through a chelation process by sequestration of metallic ions by multiple organic ligands. As such, multiple coordination bonds form between a ligand motif and the sequestered ion.^100^ The strength of metal coordination interactions varies significantly depending on the involved ligand–ion pair, with *k*_eq_ values ranging from 10^6.5^ M^–1^ for histidine–Zn^2+^ to 10^45^ M^–1^ for catechol–Fe^3+^.^72,79,81^ A widely known example of metal-coordination interactions is the complexation between catechol groups of the dihydroxyphenylalanine (DOPA) amino acids and Fe^3+^ cations in the cuticle of mussel byssal threads.^101^ Numerous studies described such mussel-inspired self-healing hydrogels based on reversible catechol–Fe^3+^ coordination. In the earliest report, self-healing hydrogels were developed by combining 4-arm PEG polymers with Fe^3+^ cations.^92^ Although this study did not investigate the injectability of the hydrogels, this strategy has been successfully employed by other groups to develop self-healing injectable hydrogels.^72,102,103^ An alternative mussel-inspired strategy involves the use of histidine–metal ion coordination for hydrogel cross-linking. However, the injectability of such hydrogel systems has only been demonstrated prior to complete gelation.^104,105^

Bisphosphonate-containing drugs, such as alendronate, are often employed as medication to treat osteoporosis. The bisphosphonate groups in these drugs exhibit a high binding affinity toward the calcium within the inorganic phase of bone. Accordingly, the strong yet reversible (*k*_eq_ ∼ 10^17.25^ M^–1^)^72,80^ nature of these coordination interactions offers a great opportunity for the design of self-healing injectable hydrogels. Because Ca^2+^ ions are abundantly present in the mineral phase of bone, this strategy has been particularly successful for the development of bone regenerative systems.^106−111^

A common coordination-based strategy involving Ca^2+^ ions is the use of alginate biopolymers. The hydroxyl and carboxylate groups within alginate chains can form complexations with Ca^2+^ ions, leading to hydrogel formation. The viscoelastic properties of such hydrogels can be easily tuned by various factors such as molecular weight of alginate chains and concentration of Ca^2+^ ions used.^29,112−114^ Although injectable systems based on alginate–Ca^2+^ coordination have been successfully developed for tissue regeneration,^115−118^ the self-healing capacity of this system from shear-induced damage has not been extensively demonstrated in literature.^72^ Nevertheless, previous work has shown that alginate hydrogels can self-heal after network disruption caused by ultrasonication employed for on-demand drug release.^119^ Similar to alginate, carrageenans exhibit a rapid and strong ion-sensitive gelation that can be exploited for self-healing hydrogel design.^120,121^ Interestingly, the coordination of carrageenans with different ions can be exploited to induce secondary, tertiary, and quaternary structures in carrageenans, yielding hydrogels with tunable properties.^122^

##### 3.1.1.3 Hydrophobic Interactions

Hydrophobic interactions play a central role in various biological processes such as protein folding.^123^ These reversible interactions are driven by nonpolar moieties or hydrophobic sections of polymer chains, which tend to minimize contact with water molecules through their aggregation. Hydrogels based on hydrophobic interactions can be formed by modification of hydrophilic polymers with hydrophobic units such as alkyl chains.^124^ Alternatively, hydrogel building blocks can be modified with hydrophobic peptides, as exploited for elastin-like polypeptides with glycine-rich and proline-rich hydrophobic domains,^125,126^ as well as by conjugation of poly(γ-*o*-nitrobenzyl-l-glutamate) to 4-arm PEG.^93^

Host–guest interactions involving hydrophobic interactions with cyclodextrin (CD) motifs as the host molecule have also been frequently exploited to construct self-healing injectable hydrogels. CD molecules are cyclic oligosaccharides which contain an interior hydrophobic cavity. Several studies have functionalized polymers (e.g., hyaluronic acid) with CD motifs as host and complementary hydrophobic moieties such as adamantane (Ad) molecules as guest motifs.^32,127−131^ This reaction has a *k*_eq_ of ∼10^5^ M^–1^.^82,83^ Such two-component systems can self-assemble into self-healing injectable hydrogels thanks to reversible CD-Ad complexation.^32,129^ Interestingly, other studies have exploited the intrinsic aromatic groups of gelatin (e.g., phenylalanine) to form reversible host–guest cross-links with CD-functionalized polymers for self-healing hydrogel design.^132,133^

##### 3.1.1.4 Hydrogen Bonding Interactions

Hydrogen bonds are dipole–dipole interactions that are responsible for a wide range of biological processes such as DNA base-pairing. A hydrogen bond involves a donor and acceptor, which are typically a hydrogen-bonded electronegative atom and another electronegative atom in its vicinity. Several strategies have been developed for the design of self-healing injectable hydrogels that rely on hydrogen bonds.^134−143^ A prominent example is the linking of ureido-pyrimidinone (UPy) units to a polymer chain via alkyl-urea spacers.^135,136^ This reaction has a *k*_eq_ of 6 × 10^7^ M^–1^ (measured in chloroform at 25 °C).^84^ In water, hydrogen bonds need to be shielded from competing hydrogen-bonding water molecules to be effective cross-linkers for hydrogels. In UPy-based hydrogels, the alkyl spacers can create a hydrophobic pocket, shielding the hydrogen-bonding UPy or urea moieties from the water molecules. This strategy has been successful for the development of self-healing hydrogels with different polymeric backbones.^137−141^ An emerging strategy for the design of self-healing injectable hydrogels is based on catechol-mediated hydrogen bonding. While catechols have traditionally been employed for hydrogel design via metal coordination interactions or quinone-based covalent bonding upon their oxidation, recent studies have demonstrated that catechol motifs can also be employed for the formation of self-healing injectable hydrogels based on hydrogen bonding.^142,143^ The complementary pairing of DNA strands is another avenue for hydrogel formation based on reversible hydrogen bonding. Although injectable^144^ or self-healing^145^ systems have been developed using this strategy, this approach has not yet been extensively employed for self-healing injectable hydrogels. These designs rely on the intrinsic capacity of DNA strands for complementary pairing between adenine (A) and thymine (T), as well as guanine (G) and cytosine (C) nucleotides. The high specificity of DNA base pairing exhibits a great potential to precisely define, at the nanoscale, the assembly of self-healing injectable hydrogels and their resulting properties.

#### 3.1.2 Dynamic Covalent Interactions

**Figure 3:**
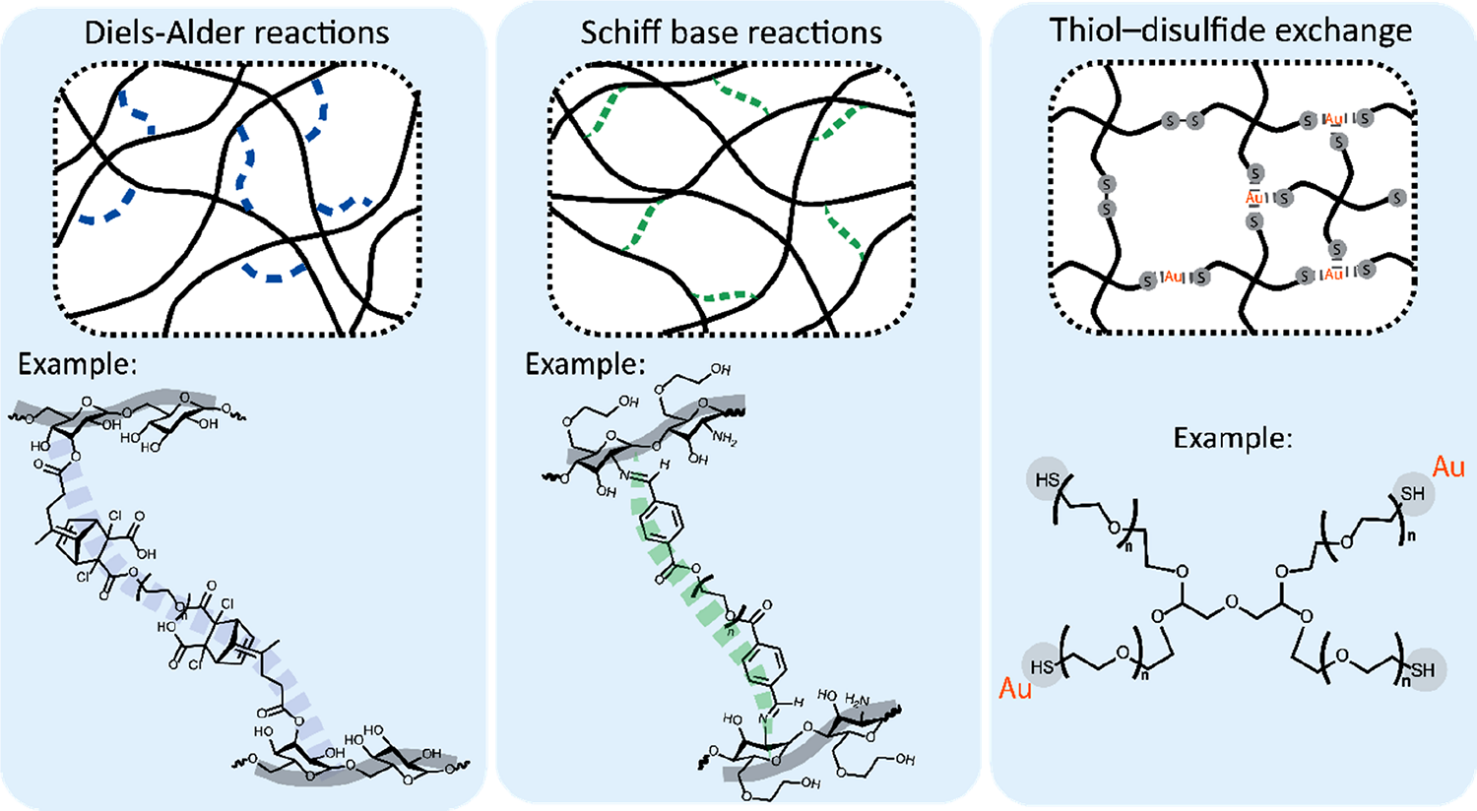
Overview of common strategies for the design of self-healing injectable hydrogels based on dynamic covalent interactions. Examples, from left to right, are based on refs (146), (147) and (148), respectively. Adapted with permission from ref (4). Copyright 2018 Wiley.

Chemical cross-linking of polymers by covalent bonds is the conventional strategy for hydrogel formation. However, these cross-links are typically static and do not exhibit the reversibility required for the design of self-healing injectable hydrogels. Therefore, dynamic covalent interactions have emerged for the formation of self-healing systems. Figure 3 provides an overview of common reactions that can be used for the design of self-healing injectable hydrogels based on dynamic covalent interactions.

##### 3.1.2.1 Diels–Alder Reactions

Diels–Alder (DA) reactions take place between a diene and a dienophile. DA reactions are described as “click-type” reactions because they are selective and catalyst-free without forming byproducts.^149^ For tissue engineering applications, the most common DA-based strategy involves combination of a polymer containing maleimide groups (as dienophile) and a polymer with furan groups (as diene).^150,151^ The reversibility of the resulting bonds relies on their ability to undergo a reverse reaction (retro-Diels–Alder reaction). This reversibility, however, is usually achieved at high temperatures,^152^ limiting the applicability of DA chemistry for the design of self-healing injectable hydrogels for tissue regeneration. The *k*_eq_ of furan and maleimide is ∼1 M^–1^ at 92.5 °C.^71,85^ This limitation has been overcome by employing fulvene-modified hydrophilic dextran and dichloromaleic-acid-modified PEG, as dienes and dienophiles, respectively.^146^ Thanks to the reversibility of their dynamic covalent cross-links, the hydrogels formed based on the DA reaction of these two components were able to self-heal at room temperature after shear or cutting-induced damage.

##### 3.1.2.2 Schiff Base Reactions

Schiff base reactions are the reaction of a nucleophilic group (e.g., amine or hydrazine) and the electrophilic carbon of aldehydes or ketones and result in the formation of reversible quasicovalent imine or hydrazone bonds. These selective reactions have been widely employed as dynamic covalent bonds to form self-healing hydrogels through mixing of two-component systems containing the reactive motifs.^153^ Their rate and capacity of self-healing is determined by the involved chemical groups and their specific *k*_eq_.^154^ For instance, for hydrazone linkages *k*_eq_ values between 10^4^ and 10^6^ M^–1^ have been reported.^86,87^ The most commonly employed Schiff base approach is the reaction between an aldehyde-functionalized polymer with the amine groups of another component. This strategy for self-healing hydrogel formation has been realized in various material systems such as hyaluronic acid–cystamine,^155^ dextran–chitosan,^156^ glucomannan–chitosan,^157^ and chitosan–cellulose–polydopamine.^158^ Owing to the fast gelation kinetics of certain Schiff base reactions, recent investigations have employed dual-barrel syringe systems to facilitate injectability followed by in situ gelation.^157,158^

##### 3.1.2.3 Thiol–Disulfide Exchange Reactions

Thiol–disulfide exchange reactions exploit the dynamic behavior of disulfide bonds in the presence of nucleophilic thiols under neutral or alkaline pH conditions, enabling the formation of reversible cross-links for self-healing hydrogels. During thiol–disulfide exchange, a sulfur atom of a disulfide bond is attacked by a thiolate anion, which is an effective nucleophile. As a result, a new disulfide bond is formed, while a new thiolate becomes available.^159^ The thiolation of polymeric building blocks can be achieved by functionalization of polymer chains with thiol groups^160−162^ as well as the reduction of disulfide bonds of proteins into thiols.^163^ Thiol–disulfide exchange reactions lose their dynamic and reversible nature upon aerial oxidation of thiolate groups, which can impede the self-healing of hydrogels. This problem can be solved by protection of reactive thiolates from aerial oxidation through capping with Au(I) or Ag(I) ions.^148,164^

#### 3.1.3 Combination of Interactions

**Figure 4:**
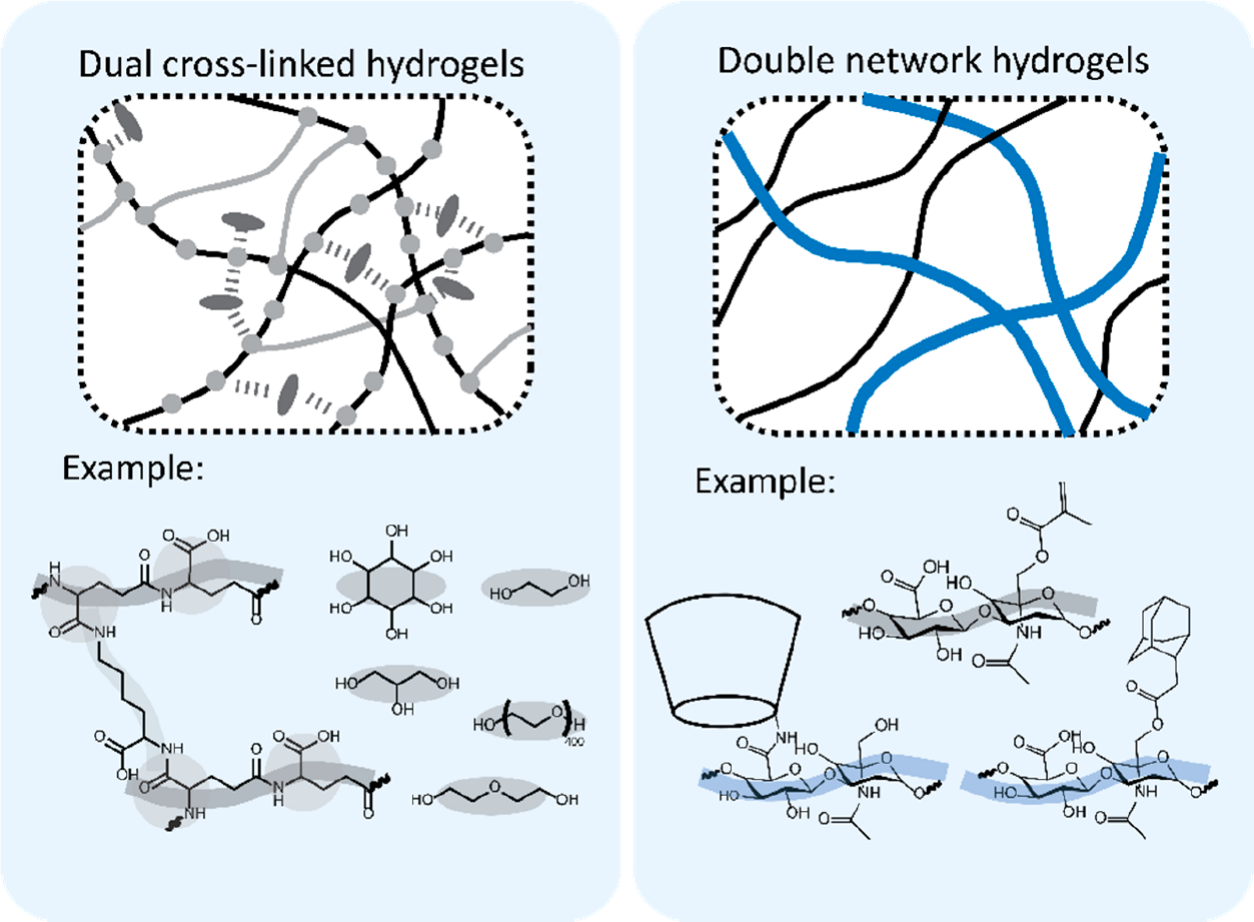
Overview of common strategies for the design of self-healing injectable hydrogels based on multiple interactions (dual cross-linked) or multiple materials (double network). Examples, from left to right, are based on refs (165) and (166), respectively. Adapted with permission from ref (4). Copyright 2018 Wiley.

Self-healing injectable hydrogels are increasingly designed by combining multiple cross-linking strategies. As shown in Figure 4, this can be achieved by (i) using different types of interactions to cross-link a single polymer network (dual cross-linked hydrogels) and/or (ii) combining two interpenetrating polymer networks which are independently cross-linked (double network hydrogels). The use of multiple interaction types or polymers can be exploited to fine-tune the mechanical properties of hydrogels and balance the trade-off between self-healing capacity and physical stability.

Noncovalent interactions are rather weak and might be sensitive to certain environmental factors (e.g., pH or ionic strength). Hence, several studies have combined multiple noncovalent interactions, typically involving electrostatic, hydrophobic, and hydrogen bonds, for the design of self-healing injectable hydrogels.^167−170^ Among the various types of dynamic covalent interactions that have been combined with noncovalent cross-links^158,171−174^ or with other dynamic covalent cross-links,^175^ significant research interest has been focused on the use of Schiff base reactions. Accordingly, imine bonds arising from these reactions have been combined with metal coordination interactions,^171−173^ hydrogen bonds,^176^ and electrostatic interactions.^174^ Interestingly, the co-utilization of metal coordination bonds involving catechol groups has been successful to endow hydrogels with tissue-adhesives functionalities.^158,173^

Another class of self-healing injectable hydrogels that benefit from multiple cross-linking modalities are double network hydrogels. These materials are composed of two interpenetrating polymer networks which are independently cross-linked. The mechanical response and stability of these materials do not solely depend on a single hydrogel network, and each network can make a distinctive contribution to the properties of the resulting double network hydrogel. A typical design of these materials exploits “weaker” noncovalent cross-links to form one network that is readily reversible to facilitate injection and rapid self-healing. The second network is cross-linked based on “stronger” covalent bonds that can provide further mechanical support. Entanglement of the interpenetrating networks can additionally contribute to the mechanical response of these materials.^177−179^ Double network hydrogels that solely rely on noncovalent cross-links can offer new possibilities for specific applications. For instance, hybrid double network hydrogels composed of synthetic and natural polymers have been synthesized using catechol-based coordination and UPy-based hydrogen bonding, which exhibit photothermal properties that are responsive to near-infrared light and pH stimuli.^180^

Naturally, the reinforcement of self-healing hydrogels with additional cross-links can be also achieved using static covalent bonds. Although static covalent cross-links based on Michael-addition reaction have been employed for the formation of self-healing injectable double network hydrogels,^166^ static covalent cross-links are not reversible and typically render hydrogels noninjectable and nonself-healing. Therefore, static covalent cross-links are commonly formed postinjection (e.g., via photo-cross-linking) for in situ stabilization of extruded materials.^181−186^

Finally, it is important to point out that many interaction types, particularly noncovalent interactions, are inherent to most materials. For example, self-assembling peptides or proteins form supramolecular hydrogels typically based on a combination of interactions such as hydrogen bonding, hydrophobic, and electrostatic forces.^187−190^ Moreover, although one interaction type might be the driving force behind hydrogel formation, engineering the properties of these systems requires holistic understanding of other interactions involved. For instance, attractive electrostatic forces between anionic and cationic gelatin have been employed for hydrogel formation. Nevertheless, at high ionic concentrations, electrostatic interactions are screened, and other interactions such hydrophobic and van der Waals forces might play a more significant role in determining properties of such gelatin-based hydrogels.^62,191^

### 3.2 Physical Design of Self-Healing Injectable Hydrogels

**Figure 5:**
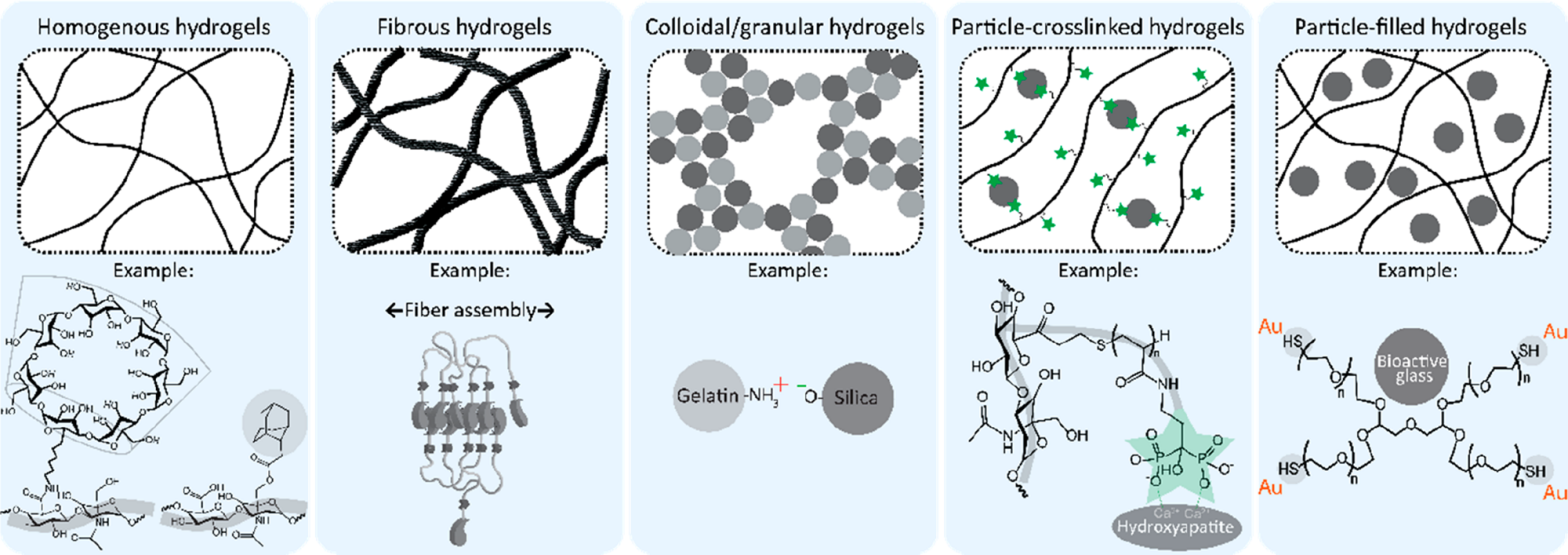
Overview of common physical forms of self-healing injectable hydrogels. Examples, from left to right, are based on refs (32), (136), (61), (110), and (192), respectively. Adapted with permission from ref (4). Copyright 2018 Wiley.

In addition to the type of chemical cross-links employed for hydrogel design, the physical form and spatial architecture of the hydrogel network largely impact the properties of the final hydrogel system. While monolithic hydrogels composed of a homogeneous 3D polymer network is the traditional strategy for the physical design of hydrogels, an increasing number of investigations employ alternative physical design approaches for structurally heterogeneous hydrogels to overcome challenges and/or endow hydrogels with unprecedented properties. An overview of physical hydrogel design strategies is provided in Figure 5. In the following sections, we discuss key strategies concerning the physical design of hydrogel networks for self-healing injectable systems.

#### 3.2.1 Monolithic Hydrogels

The most common approach for the design of hydrogels involves the use of one polymer and formation of cross-links among polymer chains. At sufficiently high polymer concentration and cross-linking (number) density, a cross-linked network of polymer chains can span the 3D space resulting in the formation of a monolithic hydrogel with homogeneous structure and gel-like behavior (*G*′ > *G*′′, frequency-independent moduli). Following this approach, various types of monolithic self-healing injectable hydrogels have been developed.^32,93,102,126,127,129,143,146,155^ In these systems, factors such as network mesh size, cross-linking density, as well as molecular weight and shape of polymeric building blocks, impact the network topology of resulting hydrogels, which in turn influence the bulk properties of hydrogels.^14,193^

#### 3.2.2 Fibrous Hydrogels

In contrast to conventional monolithic hydrogels, natural ECM in tissues is formed hierarchically from molecular building blocks commonly resulting in fibrous structures. A well-known example for this phenomenon involves fibrous collagen structures that are assembled hierarchically from molecular building blocks into fibrils, fibrils into fibers, and fibers into a 3D fibrous networks.^194^ The fibrous nature of these structures has been shown to impact mechanical (e.g., strain stiffening^195^) and biological (e.g., directional cell migration^196^) properties of these materials. Hence, research on hydrogels with a fibrous structure has gained considerable interest. Fibrous hydrogel scaffolds can be obtained by decellularizing ECM followed by reinjection.^197−201^ Alternatively, fibrous hydrogels can be assembled from collagen^202,203^ or by the bottom-up assembly of proteins into amyloids and, ultimately, fibrous hydrogels.^188,204^ A synthetic strategy comprises tuning electrostatic interactions between poly-l-lysine and self-assembling dipeptides, resulting in fibrous self-healing injectable hydrogels.^205,206^ PEG-based supramolecular hydrogels that utilize UPy motifs (discussed as hydrogen bonding groups in section 3.1.1) for dimerization and lateral stacking result in fibers, which form an entangled 3D fibrous network that is self-healing and injectable.^136^ Importantly, the properties of these fibrous hydrogel networks not only depend on the supramolecular interactions between molecular building blocks for fiber assembly but also on interfiber interactions that enable cross-link formation between the fibers.

#### 3.2.3 Colloidal and Granular Hydrogels

An emerging approach for the design of self-healing injectable hydrogels is based on the use of particles as building blocks. In this strategy, hydrogels are formed through 3D assembly or jamming of colloidal (nano)particles (nanometers to a few micrometers)^61,184,207−214^ or larger microgels (few to several micrometers).^134,215−220^ Generally, hydrogels made of colloidal particles are referred to as colloidal hydrogels, whereas those assembled from larger microgels are termed granular hydrogels. Hydrogels based on colloidal/granular building blocks have the potential to be more dynamic compared to monolithic hydrogels, which can be favorable for dynamic biological phenomena such as cell ingrowth.^207,221^ Although inorganic and/or nonswollen polymeric particles can also be employed to form colloidal hydrogels,^109,222−225^ the particulate building blocks utilized in both colloidal and granular hydrogels are usually of organic nature. Colloidal hydrogels can be obtained by the aggregation of natural organic nanoparticles. In particular, polysaccharide nanoparticles such as nanocellulose have been extensively studied in the past years for the formulation of self-healing injectable hydrogels^226−229^ or 3D printing inks.^230−234^ Another route is based on hierarchical bottom-up assembly of polymer building blocks into hydrogel particles, and assembly of these hydrogel particles into bulk colloidal/granular hydrogels. This hierarchical network design offers flexibility in terms of types of interactions and materials that can be used for the formation of self-healing injectable hydrogels. For instance, highly cross-linked hydrogels are generally not self-healing and injectable. However, smaller microgel particles made from the same material and type of cross-links may very well be self-healing and injectable.^134^ Following this approach, several self-healing injectable colloidal hydrogels have been developed based on colloidal hydrogel particles. By employing reversible noncovalent interparticle interactions, gelatin nanoparticles have been assembled, with or without other particle types, into dynamic colloidal gel networks.^61,62,109,191,207,212,218,223,235^ Covalently cross-linked hyaluronic acid-based microgels were fabricated and jammed to form self-healing injectable granular hydrogels based on reversible interparticle host–guest,^217^ hydrazone,^236^ or metal coordination interactions.^216^ Chitosan can also be aggregated into spherical microgels or nanogels to form granular hydrogels, which have been used as injectable tissue scaffolds and drug delivery vehicles.^237−240^ Recent findings suggest that altering the morphology of the microgel building blocks allows tuning of the properties of resulting granular hydrogels.^215,219^

#### 3.2.4 Particle Cross-Linked Hydrogels

In addition to their use for the formation of colloidal and granular hydrogels, particulate building blocks can also be employed for the formation of mixed polymer–particle hydrogels. To this end, attractive interactions can be exploited to enable polymer chains to bridge (nano)particles and form a percolated hydrogel network (bridging flocculation). Incorporated (nano)particles can act as fillers in polymer hydrogels to enhance their mechanical properties, as well as providing a more dynamic network structure associated with enhanced self-healing. Importantly, the mechanical response of these hydrogel materials is highly determined by their cross-linking density, as particles act as cross-linking nodes. To tune the polymer–particle interactions, polymers and/or particles can be functionalized with motifs capable of forming dynamic covalent and/or noncovalent interactions. These polymer–particle hydrogel networks have been assembled using electrostatic,^241,242^ hydrophobic,^243,244^ and coordination interactions,^106,108,110,245^ as well as molecular recognition motifs.^246^ Moreover, dynamic covalent bonds based on Schiff base,^247−250^ thiol–disulfide,^251,252^ and boronic ester^253^ have recently been employed to form these hydrogels.

#### 3.2.5 Particle-Filled Hydrogels

(Nano)particles can be included in hydrogel formulations without actively contributing to the cross-linked network. The particles can be exploited to modify hydrogel mechanical properties and often act as fillers to reinforce hydrogels. In context of self-healing injectable hydrogels, the incorporation of particles is attractive, as they can provide increased mechanical stability at rest while remaining dynamic which facilitates injection and self-healing. Besides mechanical aspects, many hydrogels for tissue regeneration are loaded with functional (nano)particles, either as drug carriers or due to their inherent antibacterial, anticancer, or pro-angiogenic action, which therefore also fall in this category. Several examples are discussed in section 5. A critical factor is a balanced distribution of particles within the hydrogel matrix to achieve uniform mechanical properties.^254^ This criterion is typically achieved by dispersing the particles within the hydrogel precursor solutions prior to gelation.^192,255^ The colloidal self-ordering of charged particles can be exploited to obtain uniform crystalline particle arrays.^254^ Alternatively, chemical strategies have been employed to allow for in situ formation of particles within the hydrogel matrix.^256,257^

## 4 Rheology Guide to Self-Healing Injectable Hydrogels

While self-healing may generally refer to the recovery of any material functionality, in the context of injectable hydrogels, it mostly refers to the recovery of mechanical properties after partial fluidization. To qualify as self-healing injectable hydrogel or 3D printing ink, a hydrogel should exhibit three key criteria: (1) A yield stress, (2) extrudability, and (3) fast recovery of the mechanical properties, e.g., viscoelasticity or yield stress after flow.^30,31,258−260^ Rotational rheology is commonly applied to characterize self-healing injectable hydrogels, however, there is currently no clear consensus which protocols are most suitable to assess these characteristics. On the other hand, certain rheological procedures are well established but may not be suitable for the intended purpose. Most notably, it should be emphasized that rotational rheology is not able to mimic the breakup and capillary flow of hydrogels during injection.^44,227,234,261^ Furthermore, there is a lack of clear rheological benchmarks that render a hydrogel injectable or printable. Here, we provide a practical guide for the characterization of self-healing injectable hydrogels using rheology and suggest which protocols and experimental setups are most suitable to assess the three individual characteristics of self-healing injectable hydrogels (a summary is provided in Box 3). For a general overview on avoiding misinterpretations in shear rheology caused by torque limits, inertia effects, surface tension, or wall slip, the reader is referred to the overview provided by Ewoldt et al.^262^

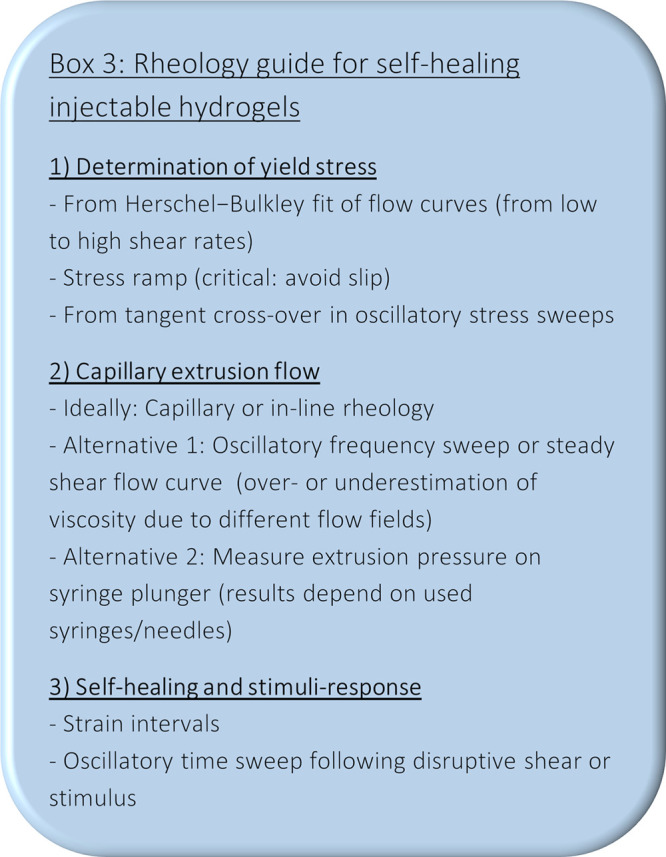



### 4.1 Yield Stress Measurement

**Figure 6:**
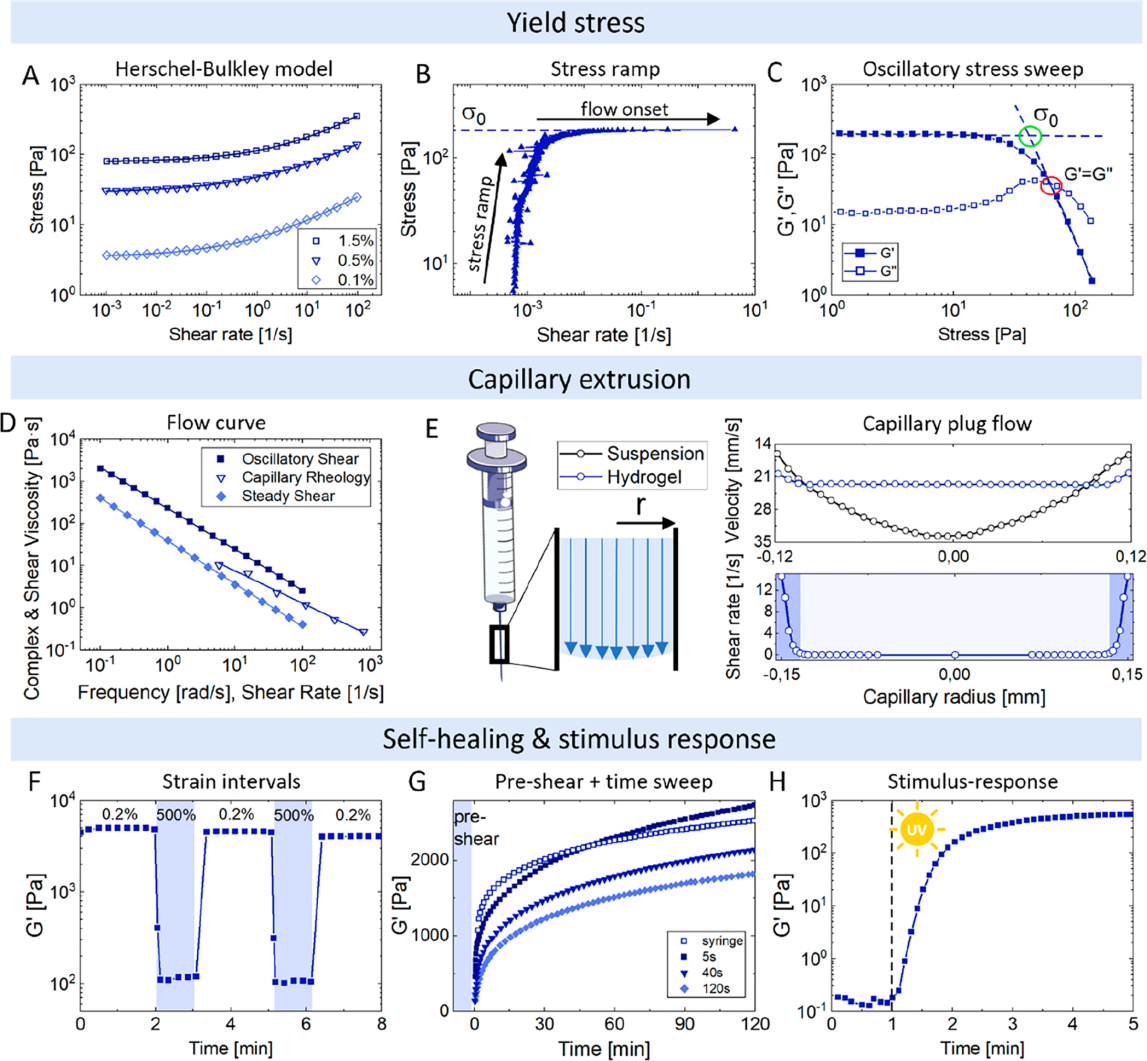
Overview of rheological protocols for the quantification of apparent yield stress, capillary extrusion, and self-healing capacity of self-healing injectable hydrogels. (A) Flow curves down to low shear rates with Herschel–Bulkley model fit. (B) Stress ramp to detect onset of material flow. (C) Oscillatory stress sweeps with apparent yield stress extraction at crossover of *G*′ tangents of the linear and nonlinear regime. (D) Flow curves of the same hydrogel obtained by oscillatory, capillary, and steady shear rheology. (E) (left) Schematic of hydrogel plug flow in a syringe needle. (right) Experimental data on hydrogel velocity and shear rate profile as a function of capillary radius compared to cell suspension. (F) Self-healing test using alternating low-high strain cycles. (G) Self-healing determined by oscillatory time sweep following preshear at a shear rate of 1000 1/s for varying periods or upon deposition of the hydrogel through a 26-gauge needle. (H) Example of stimulus-induced hydrogel strengthening to enhance mechanical hydrogel properties after extrusion. Note: Graphs show different materials. (A,C) Replotted with permission from ref (265). Copyright 2016 Elsevier. (B,D) Replotted with permission from ref (227). Copyright 2019 American Chemical Society. (E) Replotted with permission from refs (44) and (234). Copyright 2012 and 2018 American Chemical Society. (F) Replotted with permission from ref (31). Copyright 2017 American Chemical Society. (G) Replotted with permission from ref (261). Copyright 2010 Royal Society of Chemistry, (H) Replotted with permission from ref (270). Copyright 2020 Wiley.

A yield stress before and after extrusion is considered a critical factor for the mechanical stability and integrity of injectable hydrogels, as well as the shape fidelity of 3D printing inks.^30,31,258−260^ The concept and measurement of yield stresses is one of the biggest controversies in soft material rheology. Most soft materials do not have a true yield stress and will flow at sufficiently long time scales. In this case, the concept of a yield stress is an idealization trying to define the stress required to induce flow at more relevant time scales and is therefore termed apparent yield stress.^263^ As a consequence, “The” yield stress σ_0_ can be determined by several rheological protocols which may produce considerably diverging values.^264,265^ One approach involves performing rate-controlled flow curves and fitting of the Herschel–Bulkley constitutive equation (Figure 6A):^266^ σ = σ_0_ + *k*γ^*n*^, where σ is the measured stress, γ the shear rate, and *k* and *n* adjustable model parameters. To obtain ideal flow curves, this procedure is usually performed from high to low shear rates. However, structural recovery of self-healing hydrogels may interfere with the data obtained at decreasing shear rate. Therefore, it is recommended to measure from low to high shear rates and ensure full material recovery after loading, which may be guaranteed by performing an oscillatory time sweep at low strain (e.g., 0.01%) until the hydrogels have reached their equilibrium state again. Nevertheless, it may be difficult to obtain data at low enough shear rates for a good fit. Alternatively, a stress ramp experiment may be performed, where the stress is continuously increased to detect at which stress the material yields, as shown in Figure 6B. Stress ramps can be found in literature plotted as a function of shear rate, deformation, or viscosity.^227,234,267^ As these experiments are prone to wall slip they should ideally be performed using a vane or rough geometry.^268,269^ Apparent yield stresses can also be determined from oscillatory stress sweeps as shown in Figure 6C. However, there is a controversy regarding the derivation of the apparent yield stress from such strain sweeps. The crossover of *G*′ and *G*′′ is a common benchmark, which however is usually beyond material yield and overestimates the apparent yield stress. A better approximation is based on the intersection of the tangents of the linear and nonlinear regime of *G*′ (see Figure 6C), which provides values close to those obtained from Herschel–Buckley fitting.^265^ Other possibilities to determine apparent yield stresses such as stress growth or creep experiments were found impractical.^265^

### 4.2 Capillary Extrusion Flow

The assessment of hydrogel extrudability requires a major methodological optimization because the currently applied protocols are often not suitable to assess rheological characteristics of hydrogels during extrusion through a capillary. On one hand, it is common practice to claim suitability of hydrogels for injection or 3D printing based on confirmation of their shear-thinning behavior, where the terms “shear-thinning” and “injectable” are occasionally used synonymously. Shear-thinning indeed facilitates injectability through small needles as a lower pressure is required for their extrusion. However, hydrogels are almost exclusively shear-thinning, which does not automatically render them suitable for injection or printing. On the other hand, a material with an apparent yield stress and Newtonian flow, i.e., a Bingham plastic, may be injectable if it can be extruded at a reasonable pressure.^271^ Furthermore, the injectability of hydrogels is most commonly determined using rotational rheometers, which are not able to reproduce the flow profile of hydrogels during syringe or dye extrusion. Figure 6D shows flow curves of the same hydrogel obtained from three different rheological setups, namely oscillatory, capillary, and steady shear rheology, revealing considerable discrepancies regarding the measured viscosity of 1 order of magnitude. This deviation is attributed to the fact that hydrogels are structured soft materials which typically do not follow the Cox–Merz rule,^272^ i.e., the measured viscosity depends on the applied flow profile and degree of destructive shear.^273,274^ The highest viscosity was measured using oscillatory frequency sweeps in the linear viscoelastic regime where no structural breakup occurs. A medium viscosity was measured using a capillary rheology setup which most closely resembles the flow profile during extrusion. The lowest viscosity was measured using steady shear rheology, indicating destructive shear. Hence, both oscillatory and steady shear rotational rheology fail to capture the flow profile and rheology of hydrogels during capillary extrusion and result in an over- or underestimation of their viscosity, respectively.^227^ The flow profile of hydrogels during capillary extrusion was investigated by tracking incorporated fluorescent beads,^44,275^ small-angle X-ray or neutron scattering,^138,261,276^ or polarization microscopy exploiting the alignment of anisotropic particles.^234,277^ The reports all indicate that hydrogels exhibit a wide central plug flow region where the material experiences minimum shear rates, and a narrow shear zone near the capillary wall, as visualized in Figure 6E. In contrast, more liquid samples such as cell suspensions exhibit a parabolic flow profile with relative shear over the whole syringe radius, which is detrimental for cell survival.^44^ Such a plug flow cannot be accurately reproduced using rotational rheology. Hence, to truly capture the flow profile of injectable hydrogels during extrusion, the use of a capillary extrusion setup is essential.

A straightforward approach to this end involves force measurement directly at the syringe plunger upon hydrogel extrusion, allowing easy assessment and comparison of different hydrogel formulations.^31^ The disadvantage of this technique is that the force measured at the syringe plunger strongly depends on the employed syringe and needles which limits its reproducibility. In a capillary rheology setup, the pressure is measured inside the syringe before the contraction, which allows calculation of the material viscosity independent of the setup. Capillary rheology is considered the most suitable technique to determine hydrogel viscosity during injection. There are several commercial benchtop capillary rheometers available, which however are usually designed for relatively high volumes and pressures and have rarely been employed for biomedical hydrogels.^278^ Nevertheless, several viable alternatives have been proposed in literature. Lopez-Hernandez et al.^279^ combined a syringe pump with a load cell to control extrusion pressure and measure the corresponding flow rate. Bertsch et al.^227^ described a straightforward capillary rheometer setup based on inexpensive and readily available equipment, namely a syringe pump and a 3D printed syringe attachment that houses a pressure sensor and can mount different capillaries. One problem of these setups can be the relatively high sample volumes required compared to rheometers. As alternatives that require lower volumes, setups based on a micropipette modified with a stepper motor and microcontroller^280^ or microfluidics^281^ have been proposed. For 3D printing applications, Coogan and Kazmer^282^ described an elegant 3D printing nozzle containing a pressure transducer and a thermocouple, essentially upgrading the nozzle to an in-line rheometer. As an alternative to capillary rheology, Allmendinger et al.^283^ proposed a mathematical model to translate rheological data obtained by rotational rheology to extrusion flow.

Another bottleneck that may impede translation of hydrogel formulations from the rheometer to specific applications is the lack of quantitative rheological benchmarks that render a hydrogel injectable or printable. There have recently been efforts to quantify the range of the consistency index *k* and flow behavior index *n* of power law fluids.^279,284^ An interesting insight from these studies is that hydrogels require different flow behavior for administration via short syringes compared to longer catheters.^279^ We consider the establishment of such quantitative rheological benchmarks an important step to foster the future translation of self-healing injectable hydrogels.

To conclude, the commonly applied oscillatory and steady shear rheology protocols are not able to reproduce the flow profile of hydrogels during capillary extrusion. Along with the lack of quantitative benchmarks, this can strongly impede the translation of hydrogels toward biomedical applications. Assessment of injectability using rotational rheometers will probably remain the standard approach for the foreseeable future due to their widespread availability and usability. However, it should be stressed again that capillary rheology setups are more relevant to mimic injection and capture the true material behavior during extrusion flow.

### 4.3 Measuring Self-Healing and Stimulus-Response

Fast self-healing, here defined as the recovery of mechanical properties, is crucial for spatial in situ confinement of injectable hydrogels and shape fidelity of 3D printing inks.^30,31,38,258−260^ The kinetics of self-healing should be in a relevant range for the envisioned application. For ideal Maxwell fluids, relating the material relaxation τ_R_ time to the relevant observation time τ_obs_ (Deborah number De = τ_R_/ τ_obs_) provides a useful measure.^71^ However, most hydrogels exhibit a purely elastic response at small strains (frequency-independent moduli), and no relaxation time can be extracted from frequency sweeps. Instead, rheological protocols aim at measuring the transient recovery of viscosity/elasticity after breakup. The transient self-healing of injectable hydrogels can be determined by oscillatory time sweeps following destructive shear, either by short periods of high strains (e.g., 1000%), sometimes called the three interval-time-thixotropy (3ITT) test, or a preshear at high shear rates.^31,260,261^ Multiple consecutive high–low strain cycles are often performed to confirm that self-healing properties are maintained, as visualized in Figure 6F. Reported protocols for the high strain phase vary considerably in literature regarding strain values and periods, which impedes the comparability of hydrogel breakup and self-healing. Furthermore, while oscillatory time sweeps are suitable to capture the self-healing of hydrogels, oscillatory strain or shear is not able to mimic the destructive shear experienced by hydrogels during extrusion, as discussed in detail in section 4.2. Hydrogels are transported in a plug flow through capillaries and only experience structural breakup near the outer wall.^44,234,261,275^ Yan et al.^261^ convincingly demonstrated that the rate of self-healing strongly depends on the time and shear rate of the applied preshear, as shown in Figure 6G. Moreover, the authors tried to mimic the recovery following extrusion by injecting the hydrogel through a syringe directly on the rheometer, quickly lowering the geometry, and measure self-healing. While this approach is not ideal due to the lowering of the plate, it exemplifies the problem of mimicking hydrogel breakup in capillary shear because the observed self-healing kinetics differed from those observed for any rotational preshear (Figure 6G). As a consequence, self-healing kinetics reported in literature are difficult to compare as different strains or preshear treatments are employed. Furthermore, the term self-healing is used rather qualitatively and sometimes ambiguously, as there is no defined recovery threshold to justify its use. Nevertheless, measuring *G*′/*G*′′ or viscosity in oscillatory time sweeps is certainly suitable to capture the transient self-healing of injectable hydrogels following destructive shear. The destructive shear during the 3ITT test or preshear are rather exaggerated compared to the plug flow in capillaries and should therefore not impede the translation of injectable hydrogels. While many hydrogels may exhibit transient self-healing for minutes to even hours after shear, a sufficiently fast recovery within the first few seconds is probably most relevant for applications as injectable hydrogels or 3D printing inks.

There is a great interest to design hydrogels that can be mechanically reinforced after injection beyond their intrinsic self-healing capacity. Such stimuli-responsiveness can also be captured by oscillatory time sweeps while applying the stimulus, as shown in Figure 6H. For injectable hydrogels, the change in ambient temperature^285−292^ or pH^61,98,99^ in the body may be exploited to mechanically strengthen hydrogels. While the temperature can be readily adapted in most rheological setups, the variation of pH or ionic strength may require specialized geometries that allow for subphase exchange,^293^ controlled setting of hydrogels,^294^ or the use of slow-dissolving salts or acids.^61^ Magnetic hydrogels have been developed and investigated using specialized rheological setups, allowing the local heating or stiffening upon application of a magnetic or electrical field.^295,296^ A common stimulus-responsive reinforcement strategy that is particularly useful for 3D printing inks involves the application of photo-cross-linking after extrusion, which requires a transparent UV-rheology setup to capture the increase in mechanical properties.^270,297^

## 5 Applications of Self-Healing Injectable Hydrogels for Tissue Regeneration

Self-healing injectable hydrogels are at the forefront of many emerging strategies for tissue regeneration. Their main advantage is their ability to be injected locally in a minimally invasive manner followed by self-healing and recovery of their mechanical properties to guarantee in situ hydrogel confinement. In several applications, the mechanical support of self-healing hydrogels may already be sufficient to aid tissue regeneration, e.g., after myocardial infraction.^33,298,299^ spinal cord injuries,^300,301^ or vitrectomy.^302^ It is important to note that rheological requirements for injectable hydrogels may be different for delivery by a short syringe compared to a long percutaneous catheter.^279,303^ Lopez Hernandez et al.^279^ provided an insightful analysis of hydrogel administration in syringes vs catheters, including quantitative boundary limits for the consistency index *k* and flow behavior index *n* for both administration routes. Another key advantage of self-healing injectable hydrogels is their moldability to patient-specific tissue defects, which allows for personalized interventions. Particularly in combination with noninvasive imaging techniques, the volume, location, and even stiffness of administered hydrogels can be adapted to the patient-specific injury.^35,304−307^ Self-healing hydrogels may be loaded with bioactives to further promote tissue regeneration or therapeutics to combat diseases. The fast self-healing allows local confinement of hydrogels and incorporated compounds, resulting in a higher and more sustained effective dose and reduced off-target side effects.^38,41,42,308^ The retention at the injection site and biodistribution of injected hydrogels is an aspect that is often neglected. Schotman and Dankers^38^ recently provided an insightful review on this issue, pointing out the importance of hydrogel mechanical properties and degradation, administered volume, as well as biological factors such as tissue contractions and hydrogel-tissue affinity. Drug release kinetics of therapeutics from hydrogels depend on the hydrogel pore size, drug size, and solubility, and drug–matrix interactions.^14,42,309,310^ Drug release mechanisms can range from simple Fickian diffusion^310,311^ to sophisticated stimuli-responsive or on-demand release systems.^312−315^ Hydrogels equipped with complementary oligonucleotide sequences or antibodies have been proposed as reloadable depots that can actively capture systemically administered drugs from the bloodstream.^316−319^ The longevity of hydrogels is also exploited for the local delivery of excitable particles for repeated photothermal or brachytherapy against cancer.^320−322^

Hydrogels for tissue regeneration are often designed to provide an ideal physicochemical environment for cell ingrowth and subsequent tissue regeneration in terms of, e.g. porosity, cell adhesion ligands, and viscoelasticity.^18,323−325^ Because of the plug flow in capillaries (Figure 6E) hydrogels are suitable for the delivery of live cells, as embedded cells experience a reduced shear stress compared to suspensions.^43,44,326−328^ Cell survival rates are also maintained in longer catheters.^329,330^ Hydrogels can thus be used as carriers for live stem or progenitor cells to stimulate tissue regeneration^326,328,331−337^ or cell encapsulation therapy, i.e., the administration of genetically engineered cells which release regenerative growth factors.^338,339^ The mechanical properties of hydrogels significantly affect the recruitment and differentiation local cells. For instance, soft hydrogels have been found to promote adipogenesis, while stiffer hydrogels induce osteogenesis of stem cells.^112,340^ Hydrogels are also increasingly designed to specifically recruit local immune cells and exert immunomodulatory effects, e.g., for cancer immunotherapy.^341−344^ There are many self-healing injectable hydrogel formulations currently at a preclinical or clinical stage. Interesting perspectives how to foster their translation are provided by Correa et al.^16^ and Øvrebø et al.^345^

**Figure 7:**
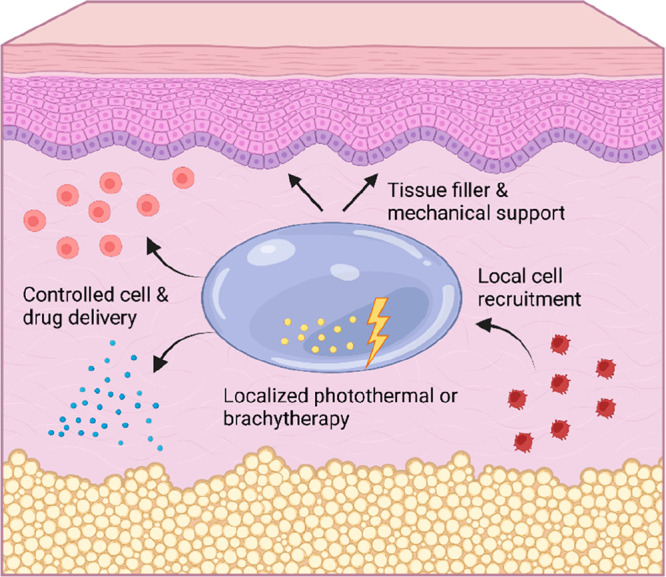
Schematic overview of tissue regeneration strategies employing self-healing injectable hydrogels.

To conclude, self-healing injectable hydrogels may be exploited for several tissue regeneration strategies, either as “empty” hydrogels for mechanical support, for controlled delivery of drugs or cells, for repeated localized radiation therapy, or for local cell recruitment. A schematic overview of tissue regeneration strategies using self-healing injectable hydrogels is provided in Figure 7. Tissue regeneration strategies for specific tissues are discussed in more detail in the following subsections.

### 5.1 Cardiac Tissue Regeneration

**Figure 8:**
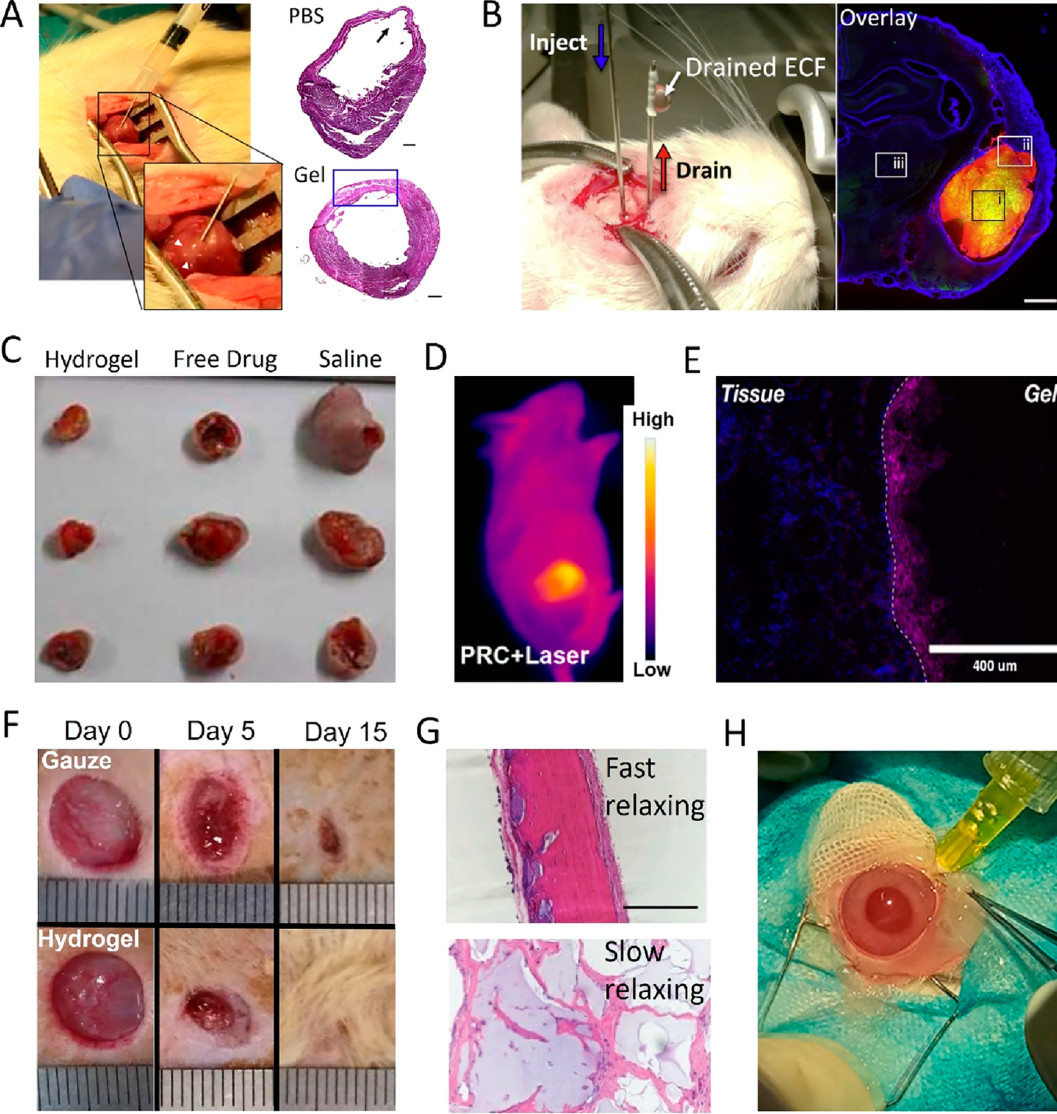
Overview of applications of self-healing injectable hydrogels for regeneration of different tissues. (A) Cardiac hydrogel injection to provide mechanical support and prevent left ventricular remodeling after myocardial infarction compared to injection of phosphate buffered saline (PBS, scale bar = 0.5 mm). Reproduced with permission from ref (31). Copyright 2017 American Chemical Society. Reproduced with permission from ref (352). Copyright 2009 Elsevier. (B) (left) Hydrogel injection into stroke cavity with simultaneous drainage of extracellular fluid (ECF) to maintain intracerebral pressure. (right) Histological image showing the injected hydrogel in the stroke cavity (orange, scale bar = 1 mm). Reproduced with permission from ref (36). Copyright 2015 Elsevier. (C) Images of excised fibrosarcomas 20 days after intratumoral injection of doxorubicin in a hydrogel matrix, free doxorubicin, or saline. Reproduced with permission from ref (379). Copyright 2015 American Chemical Society. (D) Infrared image of an intratumorally administered injectable hydrogel containing excitable nanoparticles for photothermal therapy of hypoxia-resistant breast tumors. Reproduced with permission from ref (321). Copyright 2021 Elsevier. (E) Histological image showing the recruitment of dendritic cells (purple) by cytokine-loaded hydrogels from subcutaneous tissue. Reproduced with permission from ref (380). Copyright 2019 American Chemical Society. (F) Closure of *Pseudomonas aeruginosa* infected wounds dressed with gauze or antibacterial hydrogels. Reproduced with permission from ref (381). Copyright 2019 Elsevier. (G) Histological image of calvarial bone with hematoxylin and eosin staining three months postinjury showing mature collagen-rich bone for fast relaxing hydrogels and sparse disorganized collagen without mature bone for slow relaxing hydrogels. Scale bar corresponds to 180 μm. Reproduced with permission from ref (382). Copyright 2017 Wiley. (H) Injection of a hydrogel as vitreous substitute during vitrectomy. Reproduced with permission from ref (383). Copyright 2021 Elsevier.

Myocardial infarction, i.e., heart attack, is a leading cause of death and morbidity in modern society. More specifically, left ventricular remodeling after myocardial infraction is the main cause of heart failure.^346^ Myocardial infarction and ischemia result in an acute loss of myocardium and mechanical stability, which leads to dilation, hypertrophy, and tissue scarring, particularly around the left anterior descending coronary artery.^347,348^ Early postinfarction restraint and mechanical support can greatly reduce infarct expansion and pathological remodeling.^349^ Self-healing injectable hydrogels hold great promise to prevent such left ventricular remodeling, as they can be administered rapidly by minimally invasive surgery and provide mechanical tissue support, as visualized in Figure 8A. The potential of self-healing injectable hydrogels to mechanically support infarcted myocardium and prevent pathological ventricular remodeling has already been demonstrated in rodent, ovine, and porcine models.^37,200,358,350−357^ Stiffer hydrogels show greater efficiency in preventing ventricular remodeling, stressing the need for hydrogels that remain injectable but achieve high stiffness in situ, e.g., by secondary cross-linking strategies.^359^ Besides mechanical support, self-healing injectable hydrogels can be functionalized in various ways to support cardiac tissue regeneration. Hydrogels with antioxidative properties can reduce the oxidative stress in ischemic myocardium,^360−363^ while hydrogels loaded with growth factors, pro-angiogenic cytokines, miRNA, stem, or progenitor cells can stimulate cardiac repair.^364−373^ Hydrogels with immunomodulatory efficacy were shown to reduce postinfarct inflammatory response.^374^ Infarct type and extent of damage may vary considerably within patients. Self-healing injectable hydrogels offer the possibility to precisely tune the volume, placement, and stiffness of the used hydrogels to the patient-specific infarct. The combination of noninvasive magnetic resonance imaging and finite element simulations have proven particularly useful to visualize the affected region and predict the required tissue support, allowing for fast and personalized treatment of heart attacks.^35,304−307^ The spatial retention of injected hydrogels at the target site is often insufficiently considered. Schotman and Dankers^38^ recently addressed this issue specifically for hydrogels used to treat myocardial infarction and identified hydrogel formulation (mechanical properties, degradation, tissue affinity), therapy strategy (injection timing and volume), and cardiac pulsation as important factors determining the retention of injected hydrogels and contained therapeutics. An elegant approach toward enhanced spatial control exploits the pericardial cavity as natural mold for hydrogel delivery.^375,376^ First clinical trials confirmed that injectable hydrogels based on alginate can prevent pathological left ventricular remodeling and improve exercise capacity after myocardial infarction. However, while these hydrogels were well tolerated without severe side effects in one study,^377^ another study reported a 30-day mortality of 8.6%,^378^ underlining the need for further investigations toward safe and effective clinical applications.

### 5.2 Spinal Cord Injuries and Ischemic Stroke

The central nervous system comprises some of the body’s most fragile and surgically least accessible tissues, with still limited treatment options to date. Self-healing injectable hydrogels have the potential to overcome many issues associated with the clinical use of conventional biomaterials. In spinal cord injuries, viable axons are often left at the injury site that may allow partial axonal rewiring. Bridging of the lesion with a supportive structure considerably favors recovery, but invasive surgery or implants may be detrimental at this stage.^300^ Furthermore, implants are often not versatile enough for the wide variety of spinal cord injuries. Self-healing injectable hydrogels provide a new prospect, as they allow for minimally invasive administration and filling of patient specific spinal cord injuries. Self-healing injectable hydrogels have been successfully applied to bridge spinal cord lesions and promote neovascularization and axonal ingrowth in rodent and porcine models.^301,384,385−393^ Incorporated growth or trophic factors as well as progenitor or Schwann cells can further enhance tissue regeneration and axonal rewiring.^392,394−401^ Biomaterials for spinal cord injuries should ideally provide directional guidance to promote axonal rewiring. There are several possibilities to introduce structural anisotropy in injectable hydrogels, e.g., exploiting the flow field in the syringe or magnetic alignment.^228,234,402−405^ Rose et al.^402^ demonstrated that dorsal root ganglions show directed growth in a hydrogel with magnetically aligned anisotropic microgels. An interesting alternative is the design of electroconductive hydrogels that facilitate transmission of electrical signals between nerve cells and promote their rewiring.^249,406,407^ Besides spinal cord injuries, self-healing injectable hydrogels are also being investigated to prevent intervertebral disc degeneration^201,408−412^ or to deliver neuroprotective therapeutics to counteract neurodegenerative diseases such as Parkinson’s or Huntington’s disease.^413−417^ Hydrogels for spinal cord injection should not swell in situ, as this may increase the intraspinal pressure and lead to secondary injuries.^418^

Ischemic stroke is a major cause of death and disability with very limited treatment options to date. A stroke not only causes ischemic necrosis at the site of the infarct but also the release of ECM-degrading enzymes and reactive oxygen species which induce secondary ischemic injury and tissue scarring that impedes regeneration.^34,419^ Self-healing injectable hydrogels are considered promising candidates to limit secondary ischemic injury as they may be injected rapidly and minimally invasively across the blood–brain barrier into the stroke cavity (Figure 8B), provided that intracerebral fluid is drained at the same time to keep the intracranial pressure constant.^36^ Promising reports have already been published on the injection of hydrogels loaded with growth factors, neural progenitor cells, or erythropoietin directly in the stroke cavity to reduce secondary ischemic injury or promote angiogenesis and neurogenesis after stroke.^420−425^

### 5.3 Anticancer Strategies and Immunomodulation

Chemotherapy remains one of the major treatment strategies for cancer. However, chemotherapy relies on systemic delivery of multiple cytotoxic and cytostatic chemotherapeutics, which typically cause severe side effects.^426^ Other anticancer strategies are based on delivery of drug-loaded nanoparticles into tumor blood vessels^427,428^ or cancer immunotherapy, i.e., the targeted programming of immune cells to detect and kill cancer cells.^341−343^ All of these strategies potentially benefit from locally targeted peri- or intratumoral delivery via self-healing injectable hydrogels. Self-healing injectable hydrogels are able to spatially confine therapeutics and release them locally in a sustained manner, thereby maximizing therapeutic effects and minimizing off-target side effects. The use of injectable hydrogels for delivery of chemotherapeutics^429−438^ or nanoparticles^439−446^ has been investigated for various types of cancer. Intratumoral administration of chemotherapeutics using a hydrogel matrix prolongs their therapeutic efficacy compared to free drug administration and allows sustained tumor growth suppression, as shown in Figure 8C. Many self-healing hydrogels for intratumoral injection are designed for pH-responsive release, exploiting the acidic extracellular pH in tumors.^379,447−454^ Cancer cells are often more susceptible to short-term high-dose drug bursts than long-term constant doses. Hence, hydrogels with magnetic, ultrasound, or photosensitive release mechanisms have been designed to induce burst-type drug release triggered by external stimuli.^119,295,321,455,456^ Injectable hydrogels loaded with magnetic or excitable nanoparticles are employed to induce intratumoral magnetic hyperthermia or facilitate photothermal therapy,^295,321,457−461^ as shown in Figure 8D. Injectable hydrogels loaded with radioactive iodine-131 have been investigated for intratumoral radiotherapy (brachytherapy),^322,462,463^ while radiopaque hydrogels are used as injectable tissue markers^464−466^ or spacers to protect nearby tissues and organs.^467−470^ A key advantage is that hydrogels can be designed to remain intact over several weeks and can therefore be used for repeated periodic treatments. Because of their longevity, hydrogels modified with oligonucleotides that can actively capture chemotherapeutics with complementary sequences from the bloodstream have been proposed as reloadable depots for chemotherapeutics.^316^

Cancer immunotherapy is a fast-evolving treatment strategy triggering a patient’s own immune system to fight cancer. In checkpoint blockade therapy, immune checkpoint inhibitors are administered to block checkpoint proteins on tumor or T-cells that refrain T-cells from killing tumor cells.^471^ In chimeric antigen receptor (CAR) T-cell therapy, a patient’s own T-cells are genetically modified ex vivo and reinfused to specifically recognize and kill tumor cells.^472^ Both therapies have shown promising results against a variety of cancers but are still limited by low response rates and side effects. Self-healing injectable hydrogels can be exploited for a more targeted delivery of immune checkpoint inhibitors^473−477^ or CAR T-cells^478−482^ to achieve higher local effective doses and increase cell survival while reducing off-target side effects. The effectiveness of cancer therapies is often impeded by the immunosuppressive tumor microenvironment. Accordingly, injectable hydrogels have been designed for the delivery of immune-adjuvants^320,483−486^ or reprogramming of tumor-associated macrophages from pro-tumor (M2) to antitumor (M1) phenotype.^487−489^ Dendritic cell therapy is an immunotherapy approach wherein dendritic cells are activated in the presence of tumor antigens, whereafter the antigen-presenting dendritic cells migrate to lymph nodes to prime T-cells.^490,491^ Dendritic cells can be activated ex vivo followed by reinjection in a hydrogel matrix or may be administered in a hydrogel that contains a combination of immature dendritic cells, tumor antigens, and adjuvants.^492,493^ The efficacy of administered dendritic cells is often limited by poor cell survival and limited migration and homing to lymph nodes. This has triggered the approach to design biomaterials that recruit and home local dendritic cells (see Figure 8E), present tumor antigens, and trigger dendritic cell migration to lymph nodes to induce T-cell priming.^341,494−496^ Self-healing injectable hydrogels have been successfully designed for the enrichment and activation of dendritic cells and induction of specific and protective antitumor immunity.^205,380,489,497−502^

### 5.4 Wound Healing and Soft Tissue Regeneration

Impaired wound healing, wound infections, or development of chronic wounds are a major cause for complications following surgery or injury. Wound healing involves multiple hemostatic, inflammatory, catabolic, and anabolic processes that occur at time scales from minutes to months.^503^ Because of their ability to reside at the place of injury, multifunctional hydrogels with hemostatic, antioxidative, antibacterial, pro-angiogenic, and epithelializing effect have been developed that support wound healing over several healing stages.^381,504−512^ Thereby, wound closure can be accelerated compared to traditional gauze, as shown in Figure 8F. Ma et al.^513^ described an elegant multilayered injectable hydrogel able to sequentially deliver different bioactive substances in three different stages of wound healing. Czuban et al.^319^ designed a reloadable hydrogel that is able to capture and activate antibiotic prodrugs.^319^ A common approach to promote wound healing is the incorporation of functional ions or ion nanoparticles with antibacterial^514−520^ or pro-angiogenic effects.^520−524^ Recent studies have investigated the ability to modulate the inflammation processes using self-healing injectable hydrogels. For instance, fibroblast migration to the wound site can be promoted by increased hydrogel stiffness,^525^ while macrophage polarization may be modulated by high molecular weight hyaluronic acid^523^ or delivery of exosomes.^526^

Articular cartilage has a poor healing capacity due to its avascular, aneural, and nonlymphatic nature and inherently low density of chondrocytes.^527^ Accordingly, self-healing injectable hydrogels are designed to mimic native cartilage and deliver stem or progenitor cells or mature chondrocytes to promote cartilage regeneration.^197,290,528−532^ More recently, hydrogels with immunomodulatory function are investigated to enhance local stem cell recruitment and chondrogenesis.^533−535^ The chondrogenic differentiation of stem cells as well as collagen synthesis by chondrocytes is strongly affected by the presence of cell-binding ligands^536,537^ and hydrogel stiffness, with an optimum reported around 1000 Pa.^538−540^ Hydrogels loaded with anti-inflammatory drugs and cartilage regenerative properties have also been investigated for the treatment of osteoarthritis and osteoarthrosis.^541−543^

Regarding muscle regeneration, most studies addressed the regeneration of ischemic myocardium as discussed in section 5.1. For skeletal muscles, only a few studies have been dedicated to the design of self-healing injectable hydrogels as cell carriers or scavenging of reactive oxygen species to support muscle regeneration,^156,544,545^ particularly in context of avoiding muscle loss following limb ischemia.^546−548^ Wu et al.^317^ proposed a reloadable hydrogel depot that is able to capture PEG-modified growth factors from the bloodstream to treat limb ischemia. Chang et al.^549^ demonstrated the use of a magnetic injectable hydrogel that can be exogenously actuated to stimulate muscles and avoid muscle degeneration.

Soft tissue defects may arise following surgical resections, severe trauma, or lumpectomy and require soft tissue fillers for adipose tissue regeneration. Adipose tissue regeneration recently gained particular interest due to the increased number of cosmetic adipose tissue reconstructions. Autogenous lipofilling is still the gold standard, but this procedure is often impeded by low adipocyte survival and fast fat resorption.^550^ Furthermore, currently used soft tissue fillers often show side effects like inflammation, fat necrosis, or fibrosis and show poor adipocyte differentiation and survival.^550−552^ Currently, hydrogels from novel biomaterials^553−555^ or decellularized adipose tissue^198,199^ are considered as self-healing injectable soft tissue fillers with enhanced biocompatibility, improved fat retention, and promoted adipocyte differentiation and survival. Regarding hydrogel design for adipose tissue regeneration, hydrogel stiffness, and viscoelasticity should closely match native adipose tissue to enhance adipogenesis.^112,340^

### 5.5 Bone Regeneration

Critical size bone defects that cannot heal autonomously and require clinical intervention can occur due to traumatic injury, tumor removal, degenerative diseases, or congenital defects. Bone regeneration using bioceramics, allografts, or autografts remains the common strategy for in vivo bone regeneration in the clinic. However, these treatment options are invasive and associated with the risk of infection and morbidity at the donor site.^556,557^ These drawbacks stress the need for novel bone graft materials such as osteocompatible self-healing injectable hydrogels that (i) can be administered in a minimally invasive manner to fill patient-specific defects, (ii) promote bone healing also for larger defects, and (iii) do not need to be surgically removed.^558^ Several reports have been published on self-healing injectable hydrogels to fill bone defects and support healing,^559−561^ often in combination with incorporated drugs, ions, growth factors, stem cells, or microRNA to further stimulate bone regeneration.^212,218,561−568^ A significant challenge of hydrogels for bone regeneration relates to their limited mineralization ability, i.e., the precipitation of calcium and phosphate ions as hydroxyapatite crystals. The most common strategy to promote bone mineralization is the incorporation of calcium phosphate or hydroxyapatite particles.^562,569−571^ Another common approach entails the incorporation of bioactive glasses in hydrogels due their bone regenerative capacity through apatite formation and ion release, as well as their pro-angiogenic activity.^109,572−574^ Other strategies comprise soaking of hydrogels in saturated calcium phosphate solutions, incorporation of enzymes that catalyze bone mineral deposition, or incorporation of matrix vesicles.^575^ The recently recognized effect of hydrogel viscoelasticity on cell activity and differentiation shows particular promise for bone regeneration. Fast relaxing hydrogels were shown to promote osteogenic differentiation of human mesenchymal stem cells^112,576^ and enhance bone regeneration in vivo,^382^ as visualized in Figure 8G.

### 5.6 Vitreous Substitute and Ocular Delivery

Vitrectomy, i.e., the removal of parts or the entire vitreous humor, is a common ophthalmological procedure for the removal of vitreous hemorrhages and floaters or the treatment of retinal detachment, macula, or diabetic retinopathy. There has been a trend to perform vitrectomies in a less-invasive manner using syringes (small gauge vitrectomy).^577^ The removed vitreous humor is traditionally replaced by a gas or saline to maintain intraocular pressure.^302^ More recently, self-healing injectable hydrogels have emerged as long-term vitreous substitutes which more closely resemble the mechanical and diffusive properties of vitreous humor (see Figure 8H).^278,383,578−582^ Injectable hydrogels for vitreous substitution should not only exhibit suitable mechanical properties but also long-term transparency and similar refractive index values as vitreous humor. Vitrectomy of large parts of the vitreous humor can impede ocular oxygen homeostasis, which causes oxidative stress and cataract formation. Antioxidant-loaded hydrogels can prevent such postvitrectomy cataract formation.^583^

Eye drops are facile topical ocular delivery systems, but they often fail to convey effective doses to the interior eye due to rapid dissolution and physiological barriers.^584^ Self-healing injectable hydrogels have been employed for the intraocular administration of CAR T-cells to treat retinoblastoma^585^ or prevent neovascularization by delivery of antivascular endothelial growth factors.^586−592^ Ocular neovascularization can occur following infection or trauma and is a major cause of macular degeneration.^593^ Self-healing injectable hydrogels are able to provide a long-term continuous effective drug dose required to halt degenerative eye diseases.^594^ To prolong drug delivery periods, drugs are occasionally encapsulated in biodegradable microspheres dispersed in the hydrogel matrix, thereby pushing drug release periods to up to 6 months.^595−598^ Other strategies to prevent macular degeneration entail the injection of antioxidative hydrogels^360,599^ or delivery of stem or progenitor cells.^600−603^ Promising results were also obtained by cell encapsulation therapy, i.e., the administration of hydrogels containing genetically engineered cells which continuously secrete antivascular endothelial growth factors to provide long-term effects against ocular neovascularization.^338,339^

## 6 Self-Healing Injectable Hydrogels for 3D (Bio)printing

**Figure 9:**
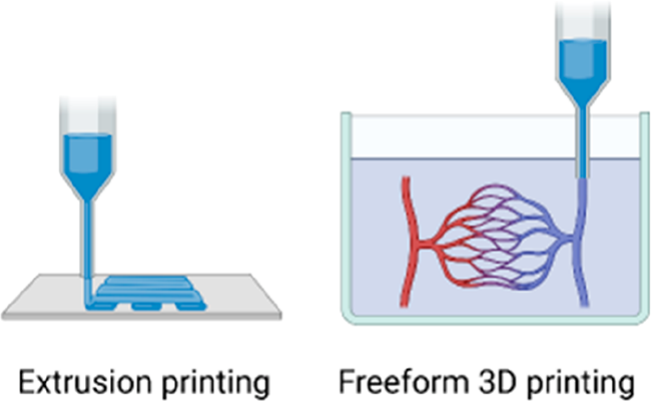
Schematic overview of applications of self-healing injectable hydrogels as 3D extrusion printing inks and/or support matrices in freeform 3D printing.

3D (bio)printing is one of the fastest evolving fields in biomedical engineering. 3D printing refers to the use of additive manufacturing, in context of hydrogels mostly extrusion printing, for the automated and computer assisted biofabrication of complex structures. Hydrogels or hydrogel precursors are commonly used inks, also called bioinks when loaded with cells^604^ or living inks when containing bacteria.^605^ In addition, self-healing hydrogels are also frequently used as support baths in freeform 3D printing. Therein, an ink is printed into a support matrix that temporarily fluidizes followed by self-healing and spatial confinement of the printed structure, allowing for almost unlimited structural freedom (see Figure 9).^49,606^ There have been several recent reviews focusing on 3D (bio)printing and its promises in biomedical engineering.^46−48,258,259,607−610^ Hence, we will herein focus on the printability of self-healing injectable hydrogels and their use as 3D extrusion printing inks or as support matrix in freeform 3D printing to obtain complex tissue and organ-like structures.

### 6.1 Hydrogel Printability

The ability of self-healing injectable hydrogels to fluidize under shear stress followed by rapid self-healing renders them versatile platforms for 3D printing inks. However, the formulation of self-healing printable hydrogels is far from trivial. Rheological tests used to assess hydrogels as 3D printing inks generally aim to determine hydrogel apparent yield stress or viscoelasticity at rest, their shear-thinning flow, and their fast self-healing, i.e., recovery of apparent yield stress or viscoelasticity after flow.^260,267^ The suitable rheological tests were discussed in detail in section 4. However, there is currently a lack of clear rheological benchmarks that a hydrogel should possess in order to qualify as 3D printing ink, strongly impeding the translation of hydrogels from the rheometer to the printer. Hereafter, we will discuss the current characterization parameters of hydrogels as 3D printing inks, followed by an outlook of how it can potentially be improved.

The suitability of hydrogels for printing is often summarized by the general term “printability”, which remains a loosely defined term that has been associated with a plethora of rheological tests and qualitative visual assessments.^258,259^ Printability is usually divided into the subcategories “extrudability”, “filament classification”, and “shape fidelity”. Extrudability refers to the ability to extrude the ink through a small needle or nozzle at reasonable pressure and is associated with a sufficiently low viscosity or shear-thinning behavior. Filament classification assesses the quality of the printed filament visually, which is ideally continuous and uniform. Filament quality is mostly an ink formulation challenge, with under-gelled inks leading to drop formation due to surface tension and overgelled inks leading to nonuniform bumpy filaments.^611−613^ Shape fidelity characterizes the ability of the printed filaments to retain their structure after printing and avoid filament fusion due to surface tension or collapse due to gravity, particularly upon printing of multiple layers. Fast self-healing kinetics (i.e., recovery of viscosity, elasticity, or yield stress) is crucial for the shape fidelity of inks.^267,614^ Ouyang et al.^612^ introduced the widely applied printability factor Pr, which characterizes the shape of squares in a printed grid according to Pr = *L*^2^/16*A*, where *L* is the perimeter and *A* the area of the squares in the grid. An ink that is printed as smooth filament and fully retains its structure after printing would result in near-perfect squares (Pr = 1), while under-gelled inks lead to filament fusion and round shapes due to surface tension (Pr < 1) and overgelled inks lead to irregular shapes (Pr > 1). Although Pr provides a certain mean of quantification for hydrogel printability and allows comparison of different inks, it is not fully clear which rheological ink parameters favor a Pr ≈ 1. Furthermore, the used printers, nozzles, and print settings largely vary among laboratories, impairing a universal comparability of Pr.

Hence, a major bottleneck in the development of hydrogels as 3D printing inks is the lack of clear rheological benchmarks. As most hydrogels are shear-thinning power-law fluids, a straightforward approach to define a “printability window” is to find the range of the consistency index *k* and flow behavior index n that facilitate printing. For instance, Liu et al.^284^ found an improved printability for *k* ≈ 1000–1800 Pa·*s*^*n*^ and *n* ≈ 0.15–0.3. While this approach facilitates to predict printability based on simple rheological experiments, it remains unclear how the data obtained by shear rheology translates to the hydrogel flow in printing nozzles. As discussed in detail in Section 4, the capillary plug flow of hydrogels cannot be adequately reproduced by shear rheology and can yield misleading flow curves.^227^ Furthermore, the actual shear stress and therefore the viscosity in the printing nozzle are often unknown. A potential solution for this problem is based on the use of nozzles with an incorporated pressure sensor that act as in-line capillary rheometers.^282^ X-ray or neutron scattering techniques can be applied to capture the structure of hydrogels during flow or their self-healing kinetics after printing.^261,276,615^ Alternatively, numerical simulations can help to understand the shear stress and viscosity of hydrogels during 3D printing.^220,284,616^

Overall, the field of 3D printing has emerged very rapidly in the past decade, and the technological advance has partially outpaced the fundamental understanding of many involved physical processes. As a consequence, the design of self-healing hydrogels for 3D printing is currently hampered by a lack of objective quantitative parameters associated with printability and often relies on a time-consuming trial-and-error approach. As specific measures to improve development, translation, and universal comparability of hydrogels designed for 3D printing, we recommend (i) the use of more suitable rheological tests (capillary or in-line rheology), (ii) establishing specific rheological benchmarks that determine printability, and (iii) increased efforts to understand the structuring of hydrogels in printing nozzles and recovery thereafter.

### 6.2 Self-Healing Hydrogels for 3D (Bio)printing of Complex Tissue Constructs

3D (bio)printing of self-healing hydrogels allows the fabrication of tissue mimetics or organoids as implantable biomaterials, tissue engineering scaffolds, or in vitro models to test novel drugs or therapies in a physiologically relevant environment without the need for animal experimentation.^46,47,617^ Compared to other manufacturing techniques, 3D bioprinting offers a more precise control over 3D structure and spatiotemporal distribution of materials, cells, and/or bioactive molecules. As discussed above in section 4, hydrogels are generally conveyed in a plug flow in 3D printing nozzles (Figure 6E), and regions of high shear forces and structural alignment are therefore often limited to the outer boundary layer.^44,227,234,261,275^ The wide plug flow with limited shear rate in the center facilitates cell survival in bioinks compared to suspensions.^43,44,326−328^ In fact, most detrimental to cell survival is the extensional flow at the entrance of the syringe rather than wall shear stresses.^43^ Nevertheless, highly concentrated inks can impede cell survival due to an increase in shear stresses.^43,612,618^ To further preserve cell viability, other parameters like printing temperature and time have to be considered. A detailed overview how to optimize bioinks for cell survival is provided by Rutz et al.^619^

**Figure 10:**
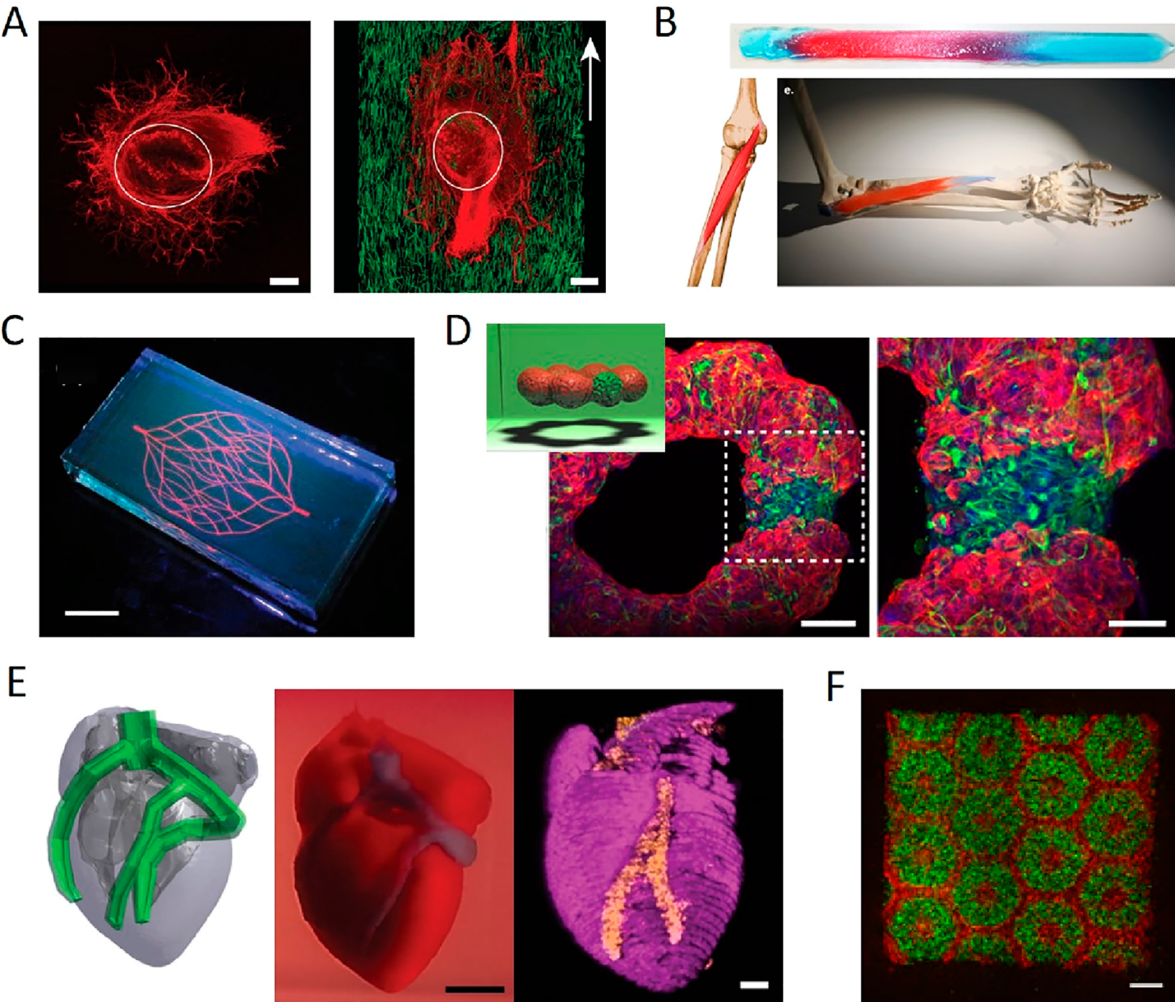
Overview of self-healing injectable hydrogels to obtain complex tissue constructs and organ-like structures. (A) Dorsal root ganglions (red) in isotropic fibrin hydrogels (left) and with anisotropic magnetically aligned microgels (right, green) promoting directed neurite growth. Reproduced with permission from ref (402). Copyright 2017 American Chemical Society. (B) Multimaterial printing of a human tendon muscle replacement with gradient mechanical properties and rigid UV-curable contact points (E-MAX 904, blue) and elastic alginate/polyacrylamide composite (red). Reproduced with permission from ref (629). Copyright 2017 Elsevier. (C) Freeform 3D printing of a sacrificial ink in a UV-curable hydrogel matrix allows omnidirectional printing of complex hollow vasculature structures (scale bar = 10 mm). Reproduced with permission from ref (606) . Copyright 2019 Wiley. (D) Deposition of cell spheroids rich in cardiomyocytes (red, “healthy”) or rich in fibroblasts (green, “scarred”) in a hydrogel matrix to obtain complex microtissues for the study of tissue defects and drug screening (scale bar = 100 μm, zoom in = 50 μm). Reproduced with permission from ref (654). Copyright 2021 Springer Nature. (E) Personalized multicellular 3D printed heart with hollow structures and vasculature. From left: The CAD model, the printed heart in the support hydrogel (scale bar = 5 mm), and 3D confocal image showing cardiomyocytes (pink) and endothelial cells (orange, scale bar = 1 mm). Reproduced with permission from ref (639). Copyright 2019 Wiley. (F) 3D printed triculture liver model with physiologically relevant hexagonal architecture enhances morphological organization, liver-specific gene expression, and metabolic activity of hepatic progenitor cells (green, red = supporting endodermal and mesodermal cells, scale bar = 500 μm). Reproduced with permission from ref (655). Copyright 2016 National Academy of Sciences.

Despite plug flow and limited alignment, several studies have reported strategies to print biomaterials with high degree of alignment and anisotropy to direct cell growth, as shown in Figure 10A. Material anisotropy may be achieved by exploiting shear and extensional flow during printing^234,405^ or by incorporating magnetic particles that enable alignment triggered by the application of a magnetic field.^228,402−404^ Multimaterial 3D printing allows printing of spatially controlled heterogeneous structures that reflect the mechanical and structural complexity of native tissue more closely,^620−629^ as shown exemplarily for a biofabricated human tendon muscle with rigid contact points and stretchable centerpiece in Figure 10B. 3D hydrogel printing further facilitates the design of biomaterials with spatiotemporally defined patterns of cells or bioactive molecules.^628,630−633^ Material constructs with locally concentrated growth factors were shown to be more effective at promoting angiogenesis compared to materials with homogeneously distributed growth factors.^634,635^

The structural freedom of 3D bioprinting is limited by the self-support capacity of the used hydrogel as well as its self-healing kinetics. Layer-by-layer printing is thus often not suitable for printing of branched, hollow or overhanging structures as encountered for many complex tissues or organs. These structural limitations may be circumvented by printing the ink into a support matrix, which is often also a self-healing hydrogel or a suspension bath.^49,56,606^ The printing nozzle is dragged through the support matrix which temporarily fluidizes followed by rapid self-healing to confine and support the printed structure. Using this freeform 3D printing, also termed as freeform reversible embedding or fluid bath-assisted 3D printing, complex branched structures can be printed at full structural freedom beyond the technological limitations of traditional layer-by-layer deposition.^49,606,636−640^ This highly attractive feature is exploited in particular to print vascularized structures that facilitate nutrient and waste transport as shown in Figure 10C, which has been a notorious bottleneck for the design of tissue constructs.^633,641−647^ Furthermore, more liquid low-viscosity inks that are not stable during extrusion 3D printing can be employed in freeform 3D printing due to the structural support of the matrix, thereby increasing the palette of potential ink formulations.^56,648^ The printed ink can be cured followed by removal of the support matrix (support bath-enabled 3D printing). Alternatively, the support matrix may be cured (embedded 3D printing) and the printed ink may be removed (sacrificial ink) or left in place (functional ink).^649^ Despite the vast potential of freeform 3D printing, the harmonization of ink and bath rheology as well as print settings are challenging, as they all affect the yielded region of the support matrix and resulting filament deposition.^650−652^ Instead of printing an ink, cell spheroids may be deposited into the support hydrogel which then fuse into high-cell density microtissues and can be used as complex tissue models.^653^ Daly et al.^654^ have exploited this approach to create heterogeneous tissue constructs, as shown in Figure 10D. One spheroid rich in fibroblasts was incorporated in a ring of “healthy” spheres rich in cardiomyocytes to study the contractile output and electrical synchronization of scarred cardiac tissue and efficacy of miRNA treatments.

Ultimately, combining these tools of printing anisotropic or gradient structures, multimaterial constructs, vascularized networks, and multicellular microtissues allows the printing of complex tissue and organ-like structures that facilitate personal medicine and provide better cell culture models to mimic disease or drug screening. For instance, Noor et al.^639^ have demonstrated the printing of personalized cardiac patches and hearts based on ECM hydrogels, cardiomyocytes, and endothelial cells previously obtained from a patient biopsy, as shown in Figure 10E. Ma et al.^655^ have shown that hepatic progenitor cells show enhanced morphological organization, liver-specific gene expression, and metabolic activity in a 3D printed triculture model with physiologically relevant hexagonal structure with supporting endodermal and mesodermal cells (Figure 10). For a more extensive overview of the current status of tissue and organ bioprinting, the interested reader is referred to the recent overviews provided by Mota et al.^46^ and Fonseca et al.^47^ An exciting new application area, which however remains in its infancy for the time being, is intravital 3D printing. Here, biomaterials are printed in situ, either in open surgery or by hydrogel injection followed by noninvasive patterning using a near-infrared laser.^656−658^

## 7 Conclusions

### 7.1 Design Strategies

Self-healing injectable hydrogels for biomedical applications need to fulfill a wide range of design criteria. Most importantly, hydrogels that can fluidize during injection followed by self-healing require the use of reversible (i.e., noncovalent and/or dynamic covalent) chemistry. At the same time, these hydrogels should be physically stable in situ to avoid premature disintegration and allow for spatiotemporal control over hydrogel integrity and release of encapsulated therapeutics. This trade-off between injectability and self-healing capacity vs physical stability and integrity needs to be carefully balanced in the design of self-healing injectable hydrogels. Furthermore, tailoring the self-healing kinetics to the specific time scales of injection/printing applications can be challenging. The equilibrium binding constant *K*_eq_ and the bond lifetime τ_B_ provide useful chemical modulators for self-healing kinetics, while rheology provides a suitable measure for the mechanical recovery of hydrogels (see below). In the past decade, an extensive library of suitable materials and reversible chemistries for self-healing injectable hydrogels has been established. A recent trend involves the formulation of hydrogels beyond monolithic polymer-based hydrogels that provide even more degrees of freedom, most prominently owing to the use of multiple chemistries (dual cross-linked) or multiple polymers (double network). Furthermore, the urge for more realistic ECM models has pushed several new physical design strategies such as fibrous hydrogels, colloidal/granular hydrogels, and particularly combinations thereof. These mixed systems can circumvent drawbacks of monolithic hydrogels by combining “weaker” with “stronger” bonds or large polymers with small particles, thereby optimizing the balance between reversibility and physical stability required for self-healing injectable hydrogels.

### 7.2 Rheological Characterization

Rheology is the established method to characterize self-healing hydrogels for injection or 3D printing applications. It is well accepted that a self-healing injectable hydrogel should possess an apparent yield stress, be extrudable, and at least partially recover its mechanical properties after capillary flow. However, it is not yet clear which set of rheological protocols or parameters unambiguously qualify a hydrogel as self-healing and injectable. In the case of apparent yield stresses, this ambiguity is a direct consequence of the plethora of rheological techniques reported to determine “The” yield stress. We have outlined three possible experiments which produce similar stress values. Moreover, the plug flow of hydrogels in capillaries cannot be adequately reproduced by oscillatory or steady shear rheology, and respective flow curves over- or underestimate hydrogel viscosity. This methodological shortcoming impedes translation particularly of 3D printing inks from the rheometer to the printer due to a diverging flow profile in the printer nozzle. The increased usage of capillary rheology poses a straightforward solution to avoid such discrepancies. On the other hand, assessment of self-healing kinetics by oscillatory time sweeps following destructive shear is uncontested, although it is also impeded by the inability of rotational rheology to mimic the destructive shear experienced by hydrogels during capillary flow. Above all, the establishment of quantitative rheological benchmarks,which render a hydrogel suitable for a specific applications,are an important step to facilitate the translation of self-healing hydrogels for injection or 3D printing.

### 7.3 Applications in Tissue Regeneration

The main advantage of self-healing injectable hydrogels for tissue regeneration is that these biomaterials can be administered in a minimally invasive manner through a narrow needle, while the subsequent self-healing allows for spatial confinement of hydrogels and incorporated therapeutics. The past decade has therefore witnessed the emergence of self-healing injectable hydrogels as a third generation of self-healing biomaterials designed for regeneration of a wide variety of tissues. The regenerative capabilities of these hydrogels range from simple mechanical support, encapsulation,and spatiotemporally controlled release of therapeutics or cells, to multifunctional and responsive materials that fulfill a cascade of functions over the various stages of regeneration. Self-healing injectable hydrogels are particularly advantageous for administration to sites that are otherwise difficult or dangerous to access, such as the central nervous system, where injectable hydrogels can be used to bridge spinal cord lesions. Clinical indications which require a fast intervention can greatly benefit from self-healing injectable hydrogels, e.g., to prevent pathological remodeling after myocardial infarction or secondary injury after ischemic stroke. Great potential lies in the use of noninvasive imaging techniques, which can be exploited to tailor the volume, location, and even stiffness of the administered hydrogel to the patient-specific injury, allowing for personalized interventions. Self-healing injectable hydrogels can act as long-term depots for local delivery of drugs or bioactive molecules in a sustained manner. Promising results have already been achieved in cancer therapy by intra- or peritumoral injection of hydrogels for local delivery of chemotherapeutics or radio- or photothermal therapy. Hydrogels with particularly long drug release profiles could facilitate the treatment of degenerative diseases like Parkinson’s, Huntington’s, or macular degeneration. There is a clear tendency to design self-healing injectable hydrogels with increasingly complex functionalities. For instance, multifunctional or stimuli-responsive hydrogels are developed which can aid healing over multiple stages of tissue regeneration or be reloaded with systemically administered drugs that are modified to be actively captured by the hydrogel from the bloodstream. Self-healing injectable hydrogels have also been recognized as promising candidates for immunotherapy. To this end, they can promote survival of engineered CAR T-cells or locally recruit dendritic cells, present tumor antigens, and trigger dendritic cell migration to lymph nodes and T-cell priming to induce specific and protective antitumor immunity. In conclusion, self-healing injectable hydrogels show great promise for various tissue regeneration strategies. The main challenge for the near future will be to confirm these results in preclinical and clinical studies and ultimately translate them toward safe and effective treatment options that meet regulatory standards and can be marketed at large scale.

### 7.4 Applications as 3D (Bio)printing Inks and Support Baths

3D printing for biomedical engineering has evolved rapidly in the past decade, and self-healing injectable hydrogels are expected to be crucial enabling tools to foster further progress in this area. While increasingly complex tissue constructs are already being developed, fundamental physical aspects of the 3D (bio)printing process are still poorly understood. Most pressingly, understanding of ink flow in 3D printing nozzles and its assessment using rheology, imaging, or simulations is still limited. As a consequence, the complex interplay of ink formulation and print settings often relies on qualitative visual assessment and a time-consuming trial-and-error approach. To overcome these bottlenecks, we proposed (i) the use of more suitable rheological tests (capillary or in-line rheology), (ii) establishment of specific rheological benchmarks that determine printability, and (iii) increased efforts to understand the structuring of hydrogels in printing nozzles. At the same, rapid advances in printing increasingly complex multimaterial constructs, anisotropic or gradient structures, vascularized networks, and multicellular microtissues have paved the way to print more realistic tissue and organ models that facilitate the study of disease, drug screening, or organ development. Eventually, this will allow printing of personalized tissue or organ implants and reduce the need for animal experiments.
